# Tunable Thermally Activated Delayed Fluorescence from Supramolecular Polymers Toward Application in Aqueous Media

**DOI:** 10.1002/anie.202509241

**Published:** 2025-06-30

**Authors:** Nils Bäumer, Peiqi Hu, Miku Naruse, Soichiro Ogi, Zachary M. Hudson, Shigehiro Yamaguchi

**Affiliations:** ^1^ Institute of Transformative Bio‐Molecules (WPI‐ITbM) Nagoya University Furo, Chikusa Nagoya 464‐8601 Japan; ^2^ Department of Chemistry The University of British Columbia Vancouver British Columbia V6T 1Z1 Canada; ^3^ Department of Chemistry Graduate School of Science Nagoya University Furo, Chikusa Nagoya 464‐8602 Japan; ^4^ Integrated Research Consortium on Chemical Science (IRCCS) Nagoya University Furo, Chikusa Nagoya 464‐8602 Japan

**Keywords:** π‐conjugated chromophore, Aqueous self‐assembly, Social self‐sorting, Supramolecular polymers, Thermally activated delayed fluorescence

## Abstract

Thermally activated delayed fluorescence (TADF) offers great potential for application in light emitting devices and bioimaging. Supramolecular polymers can offer intriguing properties for the same applications, such as stimuli responsiveness and self‐healing owing to their dynamic intermolecular interactions. However, merging the two has remained a formidable challenge, due to the nonplanar geometry of common TADF chromophores. Herein, we overcome this challenge by utilizing a less distorted multiple resonance TADF (MR‐TADF) chromophore connected to a polymerization inducing building block. The obtained supramolecular synthon is capable of assembling in aliphatic solvents due to combined interchromophore interactions and hydrogen bonding. Within the supramolecular ensemble the long‐lived photoluminescence properties of the chromophore are maintained. Further modification of the photoluminescence properties could be achieved by using different supramolecular modulators in a social self‐sorting approach, allowing fine‐tuning of the photoluminescence lifetime and bandwidth. Notably, the extent of intermolecular interactions can switch these assemblies from kinetically to thermodynamically controlled regimes. Finally, we employ this co‐assembly strategy to move from organic to aqueous media highlighting the potential toward biological applications.

## Graphical abstract

Hitherto thermally activated delayed fluorescence (TADF) and supramolecular polymerization have been seemingly incompatible phenomena. Herein, we demonstrate multiple resonance (MR) TADF from supramolecular polymers, which can be tuned, by using a supramolecular modulator approach. Further, a supramolecular solubilization strategy allows for the transfer of these properties to aqueous media, highlighting the potential for future application.

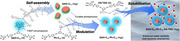

Organic chromophores capable of exhibiting thermally activated delayed fluorescence (TADF) have garnered considerable attention in recent years due to their attractive photoluminescence properties.^[^ ^1^, ^2^ ^]^ Namely, they have been extensively investigated in the context of organic light‐emitting diodes (OLEDs) due to their potential for high internal quantum efficiency.^[^ ^3^, ^4^ ^]^ Likewise, their combination of prompt as well as delayed photoluminescence makes them attractive candidates for bioimaging applications.^[^ ^5^, ^6^ ^]^ By employing time‐gated image acquisition techniques high contrast images become attainable due to the elimination of undesirable background fluorescence.^[^ ^7^ ^]^

Supramolecular polymers have equally received considerable attention for similar applications, due to their attractive dynamic properties. For instance, in‐depth control over interchromophore coupling can be achieved by tuning experimental conditions, enabling control over photoluminescence profiles.^[^ ^8^, ^9^ ^]^ Furthermore, the inherent dynamic properties of supramolecular polymers, such as their stimuli responsiveness and self‐healing capabilities, make them interesting candidates for adaptive delivery systems in the context of bioimaging and cancer therapy approaches making use of the enhanced permeation and retention (EPR) effect.^[^ ^10^, ^11^, ^12^ ^]^

However, merging the two concepts has hitherto remained a formidable challenge, with limited examples based on discrete supramolecular assemblies,^[^ ^13^, ^14^ ^]^ through‐space charge transfer mediated TADF^[^ ^15^, ^16^ ^]^ and systems with limited TADF contribution.^[^ ^17^ ^]^ TADF can be achieved, by using highly twisted chromophores composed of electron‐donating and ‐accepting moieties, resulting in a small singlet‐triplet energy gap (Δ*E*_ST_).^[^ ^18^, ^19^, ^20^ ^]^ Conversely, supramolecular polymers are typically constructed from more planar chromophore systems, due to the potential for interchromophore interactions and desirable interchromophore coupling.^[^ ^21^, ^22^, ^23^ ^]^ However, planar chromophores can also exhibit TADF properties without twisted donor and acceptor moieties as a result of multiple resonance (MR) effects (Figure 1a).^[^ ^24^, ^25^ ^]^ We recently introduced a highly flexible approach toward supramolecular polymer design, which could serve as an ideal tool to merge the two phenomena.^[^ ^26^ ^]^ Employing this strategy further enables photophysical fine‐tuning, by utilizing a supramolecular modulator approach.^[^ ^26^, ^27^ ^]^

**Figure 1:**
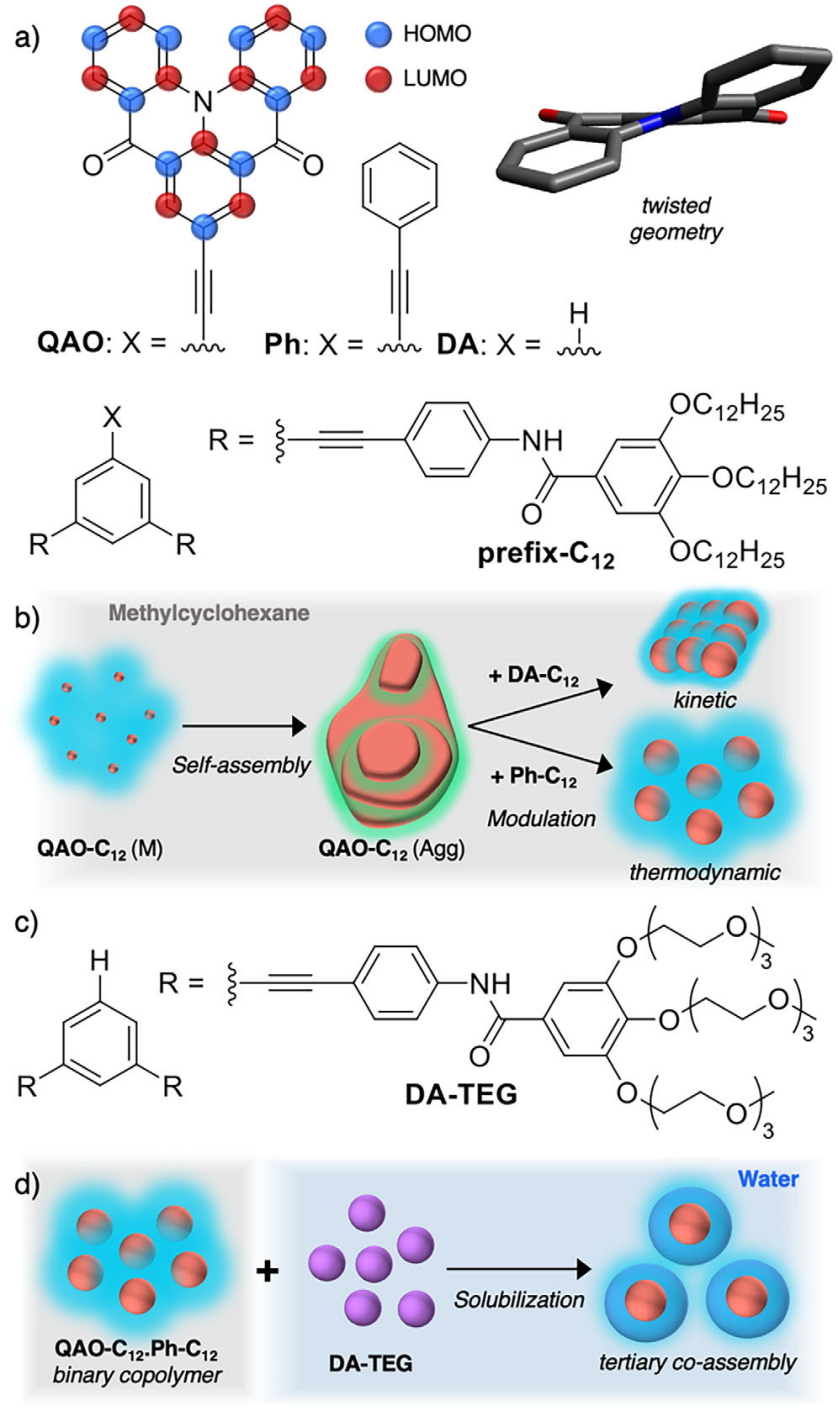
a) Chemical structures of **QAO‐C_12_**, **Ph‐C_12_** and **DA‐C_12_** including the HOMO–LUMO electron density distribution in the non‐planar **QAO** chromophore enabling MR‐TADF. b) Schematic representation of the self‐assembly behavior in non‐polar solvents including supramolecular modulation. c) Chemical structure of **DA‐TEG**. d) Schematic representation of the supramolecular solubilization.

Our molecular design consists of a self‐assembly‐inducing bent benzene diamide core with solubilizing alkoxy chains, driving supramolecular polymerization by combined dual hydrogen bonding and interchromophore interactions.^[^ ^28^, ^29^, ^30^ ^]^ MR‐TADF properties are bestowed to the ensemble by the quinolino[3,2,1‐de]acridine‐5,9‐dione (**QAO**) chromophore,^[^ ^31^, ^32^ ^]^ whereas a phenylacetylene appended (**Ph**) and an unsubstituted (**DA**) benzene diamide derivative are employed as modulators (Figure 1).^[^ ^26^ ^]^

To probe the self‐assembly behavior of **QAO‐C_12_** we conducted solvent‐dependent UV–vis absorption and photoluminescence studies (Figures 2a, S1). The absorption profile in nonpolar media such as methylcyclohexane (MCH) shows a broad, red‐shifted band in the low energy region above 400 nm (corresponding to the **QAO** chromophore) compared to moderately polar solvents, such as chloroform (Figure S1). Additionally, a red shifted shoulder and a concomitant hypochromism in the high energy regime can be appreciated, suggesting polymerization in low‐polarity media. The observation of supramolecular polymerization in low polarity solvents is consistent with other reports on structurally related compounds.^[^ ^26^, ^28^, ^29^, ^30^ ^]^ Stepwise addition of a chloroform solution of **QAO‐C_12_** to MCH leads to clear isosbestic points at 457, 391 and 345 nm, suggesting a thermodynamically controlled depolymerization process involving the monomer and one type of supramolecular assembly (Figures 2a and S2). This notion could be corroborated by thermodynamic analysis of solvent‐ as well as temperature‐dependent UV–vis absorption experiments (Tables S1 and S2; Figures S3 and S4).^[^ ^33^, ^34^ ^]^ The broadened emission profile, along with the accompanying bathochromic shift upon assembly in MCH (Figure 2b), indicate strong interchromophore coupling within the ensemble.

**Figure 2:**
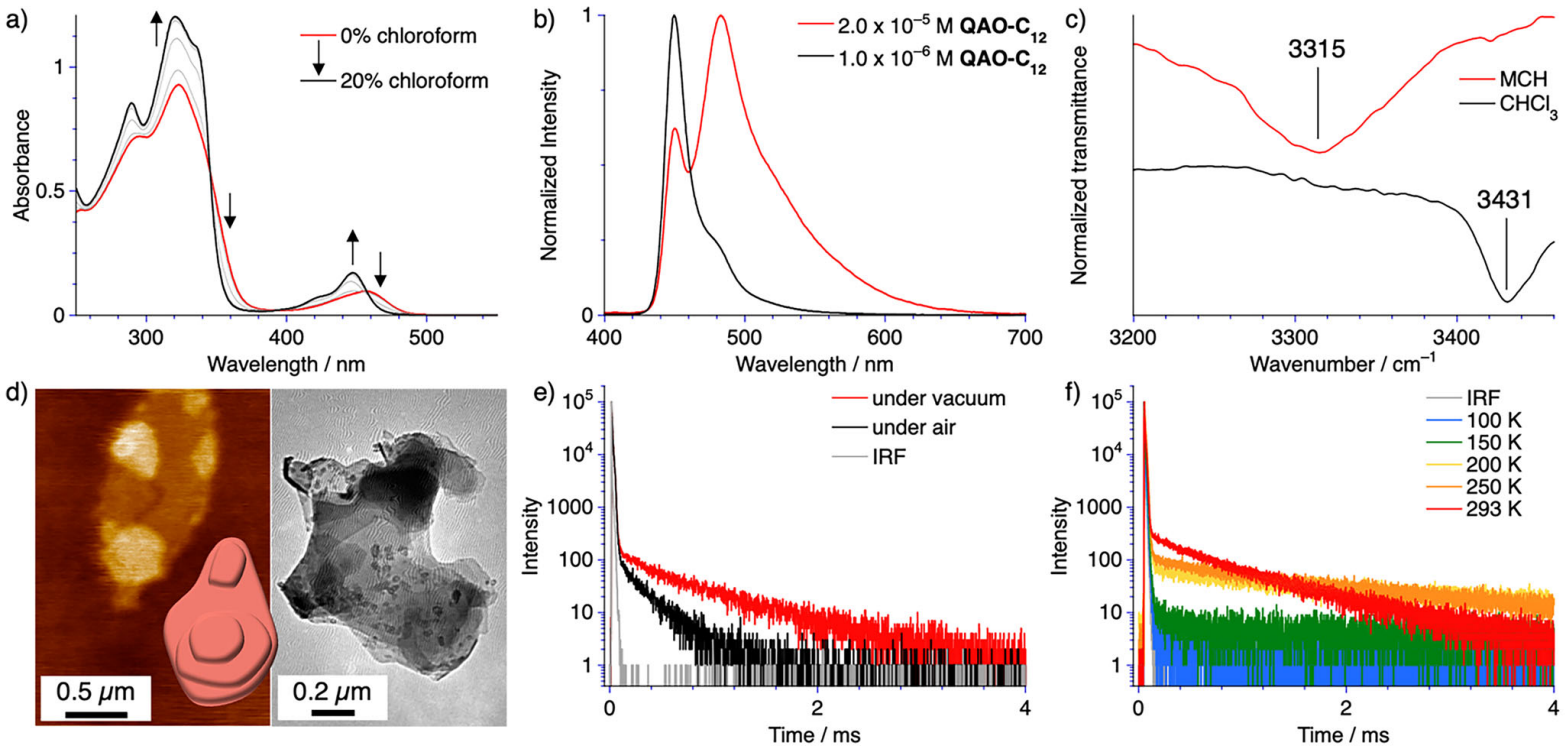
a) Solvent‐dependent UV–vis absorption spectra of **QAO‐C_12_** using MCH as self‐assembly inducing and chloroform as denaturing agent at *T* = 298 K and *c* = 1.0 × 10^−5^ M. b) Concentration‐dependent normalized photoluminescence (*λ*_ex_ = 310 nm) spectra of **QAO‐C_12_** in MCH at *c* = 2.0 × 10^−5^ M and *c* = 1.0 × 10^−6^ M c) Normalized FT‐IR spectra of **QAO‐C_12_** highlighting the N─H stretching frequency at *T* = 298 K and *c* = 5.0 × 10^−4^ M. d) AFM and TEM micrograph of **QAO‐C_12_** obtained from dropcasting (*V* = 10 µL) a MCH solution at *c* = 2.0 × 10^−5^ M (AFM) and *c* = 5.0 × 10^−5^ M (TEM). e) Photoluminescence decays (*λ*_ex_ = 420 nm, *λ*_em_ = 495 nm) of **QAO‐C_12_** in a 50 wt.% doped PMMA film under vacuum and under air. f) Temperature‐dependent long‐lived photoluminescence decays (*λ*_ex_ = 420 nm, *λ*_em_ = 495 nm) of **QAO‐C_12_** in a 50 wt.% doped PMMA film under vacuum between *T* = 100 K and 293 K.

Solvent‐dependent Fourier transform infrared spectroscopy (FT‐IR) further suggests supramolecular polymerization in low‐polarity environments. The experimentally observed N─H stretching frequency of 3315 cm^−1^ suggests intermolecular amide to amide hydrogen bonding in MCH (Figures 2c, S5). This experimental result could be corroborated by theoretical calculations suggesting a close intermolecular hydrogen bonding network between the rotationally displaced supramolecular synthons within the optimized dimer structure (Figure S6).

The resulting supramolecular morphology can be best described as hierarchical assemblies of two‐dimensional sheet‐like structures as visualized by atomic force microscopy (AFM, Figures 2d, S7) as well as transmission electron microscopy (TEM, Figures 2d, S8). This type of assembly is typically observed when individual one‐dimensional stacks experience a secondary stabilizing interaction by hierarchical organization.^[^ ^35^, ^36^ ^]^ In the case of **QAO‐C_12_** this assembly mode is likely driven by solvophobic interactions, shielding the polar carbonyl groups of the **QAO** chromophore from the nonpolar solvent medium. Additional stabilization by van der Waals interactions between the alkyl chains in the molecular periphery likely adds to the stability of the hierarchical assembly, as previously observed for related morphologies.^[^ ^37^, ^38^ ^]^

We next focused our attention on the photoluminescence properties of the ensembles of **QAO‐C_12_** in more detail using photoluminescence spectroscopy and lifetime studies (Figures 2e, S9). Increasing the concentration of **QAO‐C_12_** within PMMA films leads to a bathochromic shift of the photoluminescence maximum, along with spectral broadening as previously noted for aliphatic solutions. Moreover, a relative decrease in the long‐lived photoluminescence lifetime and quantum yield can be observed in the homopolymers of **QAO‐C_12_** (Table S3), owing to strong interchromophore coupling between adjacent chromophores within the stack. An overall decrease in the radiative decay rate constant (*k*_r_) and an increase in the non‐radiative decay rate constant (*k*_nr_) also suggest that the emission is quenched upon the aggregation of the chromophores, consistent with the observed changes in both lifetime and quantum yield (Table S3). Likewise, the full width at half maximum (FWHM) of the polymers (62 nm) is slightly larger than typical examples of MR‐TADF chromophores (20–40 nm), but remains narrow in comparison to typical charge‐transfer‐based TADF systems.^[^ ^6^, ^39^, ^40^ ^]^ Temperature‐dependent lifetime measurements corroborate that the observed long‐lived photoluminescence can be attributed to TADF (Figures 2f, S10).

In summary, the delayed fluorescence properties of **QAO‐C_12_**, such as emission wavelength, brightness, and band shape are affected by the homopolymerization, owing to strong interchromophore coupling. Recently, we have reported that photoluminescence properties of supramolecular polymers can be affected by social self‐sorting.^[^ ^26^ ^]^ In this approach, a second supramolecular synthon, a so‐called modulator, can intercalate into a supramolecular polymer, disrupting long‐range interactions, thus affecting photoluminescence. To probe if this may also be applicable to **QAO‐C_12,_** we next probed two‐component mixtures of **QAO‐C_12_** with a phenylacetylene‐appended derivative (**Ph‐C_12_**), as well as a simple benzene diamide synthon (**DA‐C_12_**, Figure 1a).

We fixed the concentration of **QAO‐C_12_** at *c* = 1.0 × 10^−5^ M and increased the concentration of **Ph‐C_12_** and **DA‐C_12_** in a stepwise fashion while monitoring the changes in photoluminescence (Figures 3a, S11). In both cases, the photoluminescence intensity increases upon increasing the equivalent concentration of the modulator. In the case of **Ph‐C_12_** the modulation process reaches a plateau at roughly 10 equivalents of the modulator, while in the case of **DA‐C_12_** significantly greater amounts are required to achieve comparable effects (Figures 3b, S11). This observation also directly translates to the long‐lived photoluminescence decays. While co‐assemblies of **QAO‐C_12_** and **Ph‐C_12_** have comparable lifetimes to those of the homopolymer of **QAO‐C_12_**, co‐assemblies with **DA‐C_12_** exhibit a decreased lifetime (Figures 3c, S12 and S13, Table S4). Moreover, in the case of modulation with **Ph‐C_12_** a larger shift of the photoluminescence maximum (Δ*λ*_em_ = 20 nm), compared to **DA‐C_12_**, could be observed (Δ*λ*_em_ = 11 nm), suggesting a partial nano‐segregation within the ensemble of **QAO‐C_12_** and **DA‐C_12_**. Nevertheless, in both copolymers the FWHM is decreased to values resembling those of the monomer, highlighting the potential of supramolecular modulation in the context of fine‐tuning MR‐TADF from supramolecular polymers.

**Figure 3:**
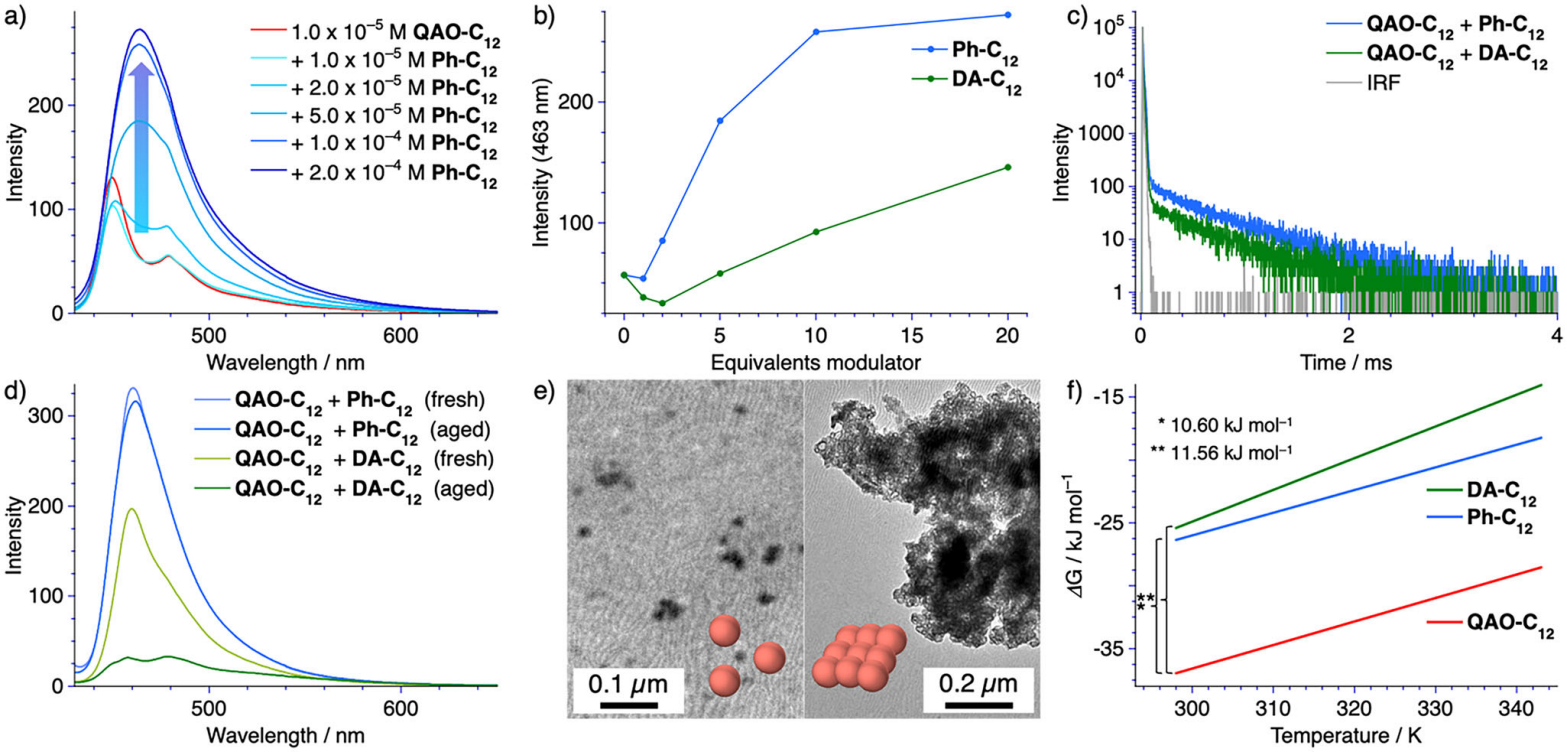
a) Photoluminescence (*λ*_ex_ = 420 nm) spectra of **QAO‐C_12_** (*c* = 1.0 × 10^−5^ M) in the presence of increasing amounts of **Ph‐C_12_** in MCH at *T* = 298 K. b) Changes in photoluminescence intensity (*λ*_em_ = 463 nm) of **QAO‐C_12_** (*c* = 1.0 × 10^−5^ M) in MCH at *T* = 298 K with increasing amounts of modulator **Ph‐C_12_** and **DA‐C_12_**. c) Photoluminescence decays (*λ*_ex_ = 420 nm) of **QAO‐C_12_**.**Ph‐C_12_** (*λ*_em_ = 475 nm) and **QAO‐C_12_**.**DA‐C_12_** (*λ*_em_ = 484 nm) copolymers in a 50 wt.% doped PMMA film (5% **QAO‐C_12_**, 45% modulator) under vacuum. d) Time‐dependent photoluminescence spectra of the co‐assemblies of **QAO‐C_12_** (*c* = 5.0 × 10^−5^ M) with **DA‐C_12_** (*c* = 5.0 × 10^−4^ M), and **Ph‐C_12_** (*c* = 5.0 × 10^−4^ M) immediately after sample preparation (fresh) and after equilibration at room temperature for 5 days (aged). e) TEM micrographs of the co‐assembled structures of **QAO‐C_12_** (*c* = 2.0 × 10^−5^ M) in the presence of **Ph‐C_12_** (*c* = 5.0 × 10^−4^ M, left) and **DA‐C_12_** (*c* = 5.0 × 10^−4^ M, right). f) Changes in the Gibbs free energy of the supramolecular homopolymers of **QAO‐C_12_**, **Ph‐C_12,_** and **DA‐C_12_** in MCH plotted against the temperature.

The use of different modulators also impacted the overall stability of the co‐assemblies. While the co‐assembled structure of **QAO‐C_12_** and **Ph‐C_12_** was found to be stable within the experimental timeframe (5 days), those of **QAO‐C_12_** and **DA‐C_12_** were found to phase separate, driven by the narcissistic self‐sorting of both compounds and subsequent precipitation of homopolymers of **DA‐C_12_** (Figure 3d, S14). This behavior could be reversed using a straightforward heating and cooling cycle, reinstating the original photoluminescence profile of the ensemble (Figure S15). This observation identifies the co‐assembly process of **QAO‐C_12_** and **DA‐C_12_** as a kinetic assembly outcome, compared to the thermodynamically controlled co‐assembly of **QAO‐C_12_** and **Ph‐C_12_**.^[^ ^41^, ^42^ ^]^

We next sought to clarify how the different modulators influence the supramolecular morphology (Figures 3e, S16 and S17). TEM and AFM analysis revealed that the co‐assembled structure of **QAO‐C_12_** and **Ph‐C_12_** is nearly identical to the spherical assemblies found for **Ph‐C_12_** in isolation under comparable experimental conditions.^[^ ^26^ ^]^ In the case of **DA‐C_12,_** such a drastic change of the morphology was not observed and instead the resulting morphology can be more accurately described as a patchy network of spherical structures, resulting in hierarchical two‐dimensional assemblies, with comparable dimensionality to those of the homopolymer of **QAO‐C_12,_** reaffirming the differences between the two modulators.

We argue that the difference between the modulators stems from the different intermolecular binding strengths between **QAO‐C_12_** with **Ph‐C_12_** and **DA‐C_12_**. To evaluate this, we probed the homopolymerization of **Ph‐C_12_** and **DA‐C_12_** (Figures 3f, S18 and S19, Tables S5 and S6). The obtained thermodynamic parameters were then employed in the supramolecular copolymerization model^[^ ^43^ ^]^ to reveal an elongation enthalpy of Δ*H*_e_ = −88.0 kJmol^−1^ and −78.0 kJ mol^−1^ for **QAO‐C_12_**/**Ph‐C_12_** and **QAO‐C_12_**/**DA‐C_12_** hetero‐elongation steps, respectively (Figures S20 and S21, Table S7). Simulating the equivalent bond concentrations (i.e., the social and narcissistic binding events) within the copolymers reveals that a **QAO‐C_12_** block structure is retained at the center of co‐assemblies with **DA‐C_12_** (Figure S21d), suggesting an increased nanoscopic segregation within the copolymer, with **DA‐C_12_** at the periphery.^[^ ^26^, ^43^, ^44^ ^]^ According to this analysis both **Ph‐C_12_** and **DA‐C_12_** can be classified as intercalators for **QAO‐C_12_**, leading to random (**Ph‐C_12_**) and “blocky” (**DA‐C_12_**) copolymer structures (see supplementary discussion 1).

To summarize, while **Ph‐C_12_** can effectively intercalate into stacks of **QAO‐C_12,_** leading to significant changes in photophysical properties and morphology, **DA‐C_12_** interacts less strongly with **QAO‐C_12,_** leading to kinetically controlled co‐assemblies with **DA‐C_12_** largely located toward the periphery of the ensemble. We argue that this nanoscopic phase separation within the copolymer of **QAO‐C_12_** and **DA‐C_12_** may be utilized to further the concept of supramolecular modulation to overcome solvation barriers.

Delayed fluorescence has been demonstrated to hold promise toward bioimaging applications.^[^ ^5^, ^6^, ^7^ ^]^ By replacing the solubilizing groups located at the periphery of co‐assembled structures, it may be feasible to employ the current systems toward application in aqueous media by using a supramolecular solvation approach. To this end, we replaced the solubilizing alkyl chains in the molecular periphery of **DA‐C_12_** with triethylene glycol giving **DA‐TEG** (Figure 1c). We then probed the homopolymerization of **DA‐TEG** in aqueous systems using a combined approach of solvent‐dependent UV–vis absorption studies and TEM. This analysis revealed a cooperative supramolecular polymerization of **DA‐TEG** at room temperature likely driven by combined hydrogen bonding and solvophobic interactions (Figure S22). The morphology of the ensembles at room temperature can be best described as spherical particles (Figure S23), likely due to the buildup of steric demand of the hydrated glycol chains upon polymerization limiting unrestricted growth despite the cooperative assembly mechanism.^[^ ^45^, ^46^, ^47^ ^]^

To avoid the formation of large, poorly dissolvable two‐dimensional **QAO‐C_12_** homopolymers, we have focused our attention on tertiary mixtures between **QAO‐C_12_**, **Ph‐C_12,_** and **DA‐TEG**, as the former two have been previously shown to form small spherical co‐aggregates (Figures 3e and 4a–d). Our sample preparation protocol encompasses an initial co‐dissolution of all supramolecular synthons in chloroform, prior to evaporation to dryness *in vacuo*. We confirmed the formation of a similar packing structure and stabilizing interactions within co‐assembled films of the three different building blocks using FT‐IR, UV–vis, and photoluminescence spectroscopies (Figures S24 and S25). Following that, the required amount of water was added directly to the film and sonication was applied at 31 kHz for no less than 10 min, until a homogenous solution was obtained. After careful optimization of the experimental conditions for solubility and photoluminescence brightness, we obtained the final conditions of **QAO‐C_12_** at *c* = 5.0 × 10^−6^ M, **Ph‐C_12_** at *c* = 2.0 × 10^−5^ M and **DA‐TEG** at *c* = 5.0 × 10^−4^ M (Figures 4a, S26–S28).

**Figure 4:**
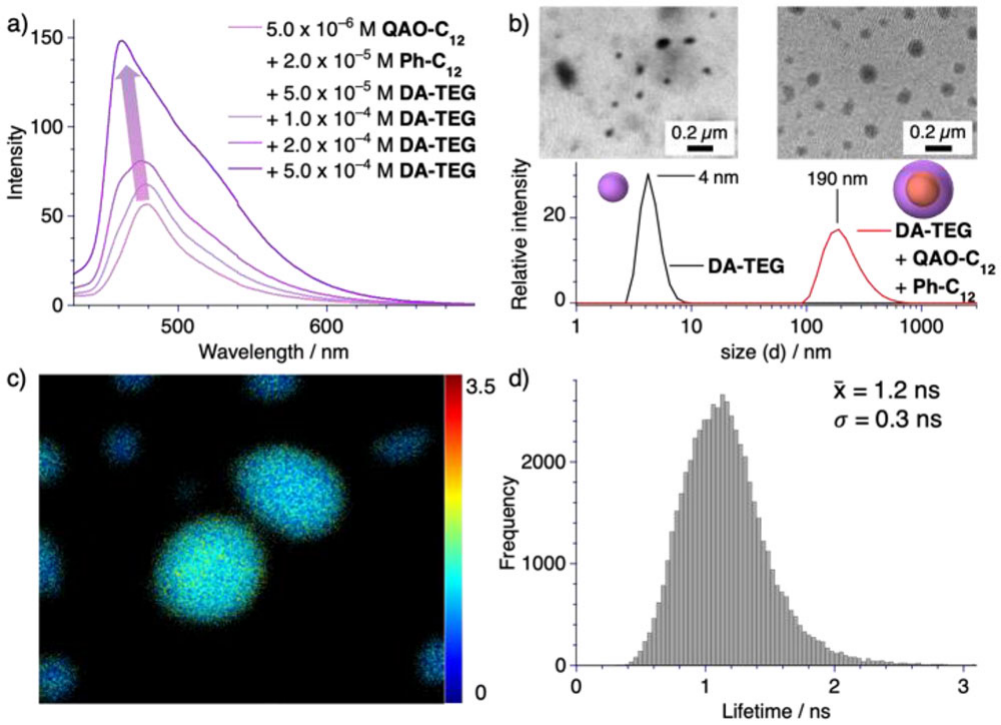
a) Photoluminescence (*λ*_ex_ = 405 nm) spectra of **QAO‐C_12_** at *c* = 5.0 × 10^−6^ M and **Ph‐C_12_** at *c* = 2.0 × 10^−5^ M in water in the presence of increasing amounts of **DA‐TEG**. b) TEM micrographs and DLS number size distribution functions of **DA‐TEG** at *c* = 5.0 × 10^−4^ M in isolation and in the presence of **QAO‐C_12_** at *c* = 5.0 × 10^−6^ M and **Ph‐C_12_** at *c* = 2.0 × 10^−5^ M in water. c,d) Fluorescence lifetime microscopy image (c) and lifetime histogram d) of **QAO‐C_12_** at *c* = 5.0 × 10^−7^ M, **Ph‐C_12_** at *c* = 2.0 × 10^−6^ M and **DA‐TEG** at *c* = 5.0 × 10^−5^ M at *T* = 298 K (*λ*_ex_ = 420 nm, *λ*_em_ = 450–550 nm).

Next, we checked the morphology of the tertiary co‐assembly between the building blocks by combined TEM and dynamic light scattering (DLS) analysis and compared the results to isolated samples of **DA‐TEG** under identical conditions (Figures 4b, S29 and S30). Both TEM and DLS analysis revealed a significant increase in the supramolecular size distribution in the presence of **QAO‐C_12_** and **Ph‐C_12_**, likely due to the encapsulation of the highly hydrophobic copolymer within a shell structure comprised largely of **DA‐TEG**.^[^ ^48^, ^49^, ^50^ ^]^ It should, however, be noted that the supramolecular solubilization by **DA‐TEG** goes beyond that of a typical amphiphile‐based surfactant, as revealed by control experiments using cetrimonium bromide (**CTAB**, Figures S28–S30). This result emphasizes the significance of the additive design. We propose that **DA‐TEG** may exhibit dual functionality in the solubilization process. Namely, during the sample preparation, a small amount of **DA‐TEG** may intercalate into the copolymer of **QAO‐C_12_** and **Ph‐C_12_** leading to increased photoluminescence brightness (Figure 4a).^[^ ^51^, ^52^ ^]^ Secondly, this partially intercalated **DA‐TEG** can serve as a binding side for further **DA‐TEG**, leading to the formation of the solubilizing shell (see supplementary discussion 2 for more detail).

Finally, we demonstrated the successful encapsulation of **QAO‐C_12_** within these co‐assemblies by fluorescence microscopy, using an excitation wavelength that exclusively targets the **QAO** chromophore (*λ*_ex_ = 420 nm). Fluorescence microscopy revealed the homogeneous distribution of **QAO‐C_12_** through all particles highlighting the efficacy of our approach (Figure S31). The extracted lifetime could be attributed to the prompt lifetime of **QAO‐C_12_** (Figures 4c,d, S32). We attribute the fact that the delayed lifetime was not observed to the overall decreased photoluminescence in aqueous media, suggesting a partial penetration of the solubilizing shell by the solvent molecules.

In conclusion, we have demonstrated that MR‐TADF from supramolecular polymers can be controlled using a supramolecular modulator strategy. By carefully controlling the social self‐sorting behavior between the chromophore‐appended building block and the modulators, the resulting ensemble could be switched between a kinetic/“blocky” and a thermodynamic/random co‐assembly outcome. Moreover, by using an additional modulator that is soluble in aqueous media, tertiary mixtures could be successfully solubilized in water, allowing investigation by fluorescence (lifetime) microscopy. Work in our laboratories is currently underway to optimize the photoluminescence quantum yield of these tertiary mixtures, as well as the shielding effect from oxygen by modifying the design of the different supramolecular synthons, with the ultimate goal of enabling bioimaging applications using time‐gated microscopy.

## Supporting Information

The authors have cited additional references within the Supporting Information.^[^ ^53^, ^54^, ^55^, ^56^, ^57^, ^58^, ^59^, ^60^ ^]^

## Conflict of Interests

The authors declare no conflict of interest.

## Supporting information



Supporting information

## Data Availability

The data that support the findings of this study are available in the supplementary material of this article.
