# Supplementary material for: Tunable Thermally Activated Delayed Fluorescence from Supramolecular Polymers Toward Application in Aqueous Media
Source: Angew Chem Int Ed Engl. 2025 Jun 30;64(34):e202509241. doi: 10.1002/anie.202509241 (PMC12363619; doi:10.1002/anie.202509241)
Supplement: Supplementary file 1 — Supporting information [file ANIE-64-e202509241-s001.pdf]

Supporting Information

Tunable Thermally Activated Delayed Fluorescence from Supramolecular Polymers Toward Application in Aqueous Media

Nils Bäumer+[a], Peiqi Hu+[b], Miku Naruse[c], Soichiro Ogi*[d], Zachary M. Hudson*[b] and Shigehiro Yamaguchi*[a,c,d]

+ these authors contributed equally to this work.

Abstract: Thermally activated delayed fluorescence (TADF) offers great potential for application in light emitting devices and bioimaging. Supramolecular polymers can offer intriguing properties for the same applications, such as stimuli responsiveness and self-healing owing to their dynamic intermolecular interactions. However, merging the two has remained a formidable challenge, due to the nonplanar geometry of common TADF chromophores. Herein we overcome this challenge by utilizing a less distorted multiple resonance TADF (MR-TADF) chromophore connected to a polymerization inducing building block. The obtained supramolecular synthon is capable of assembling in aliphatic solvents due to combined interchromophore interactions and hydrogen bonding. Within the supramolecular ensemble the long-lived photoluminescence properties of the chromophore are maintained. Further modification of the photoluminescence properties could be achieved by using different supramolecular modulators in a social self-sorting approach, allowing fine-tuning of the photoluminescence lifetime and bandwidth. Notably, the extent of intermolecular interactions can switch these assemblies from kinetically to thermodynamically controlled regimes. Finally, we employ this co-assembly strategy to move from organic to aqueous media highlighting the potential towards biological applications.

Table of Contents

Materials and Methods 3
Synthesis and Characterization 5
Additional Spectroscopy 8
References 33
Computational section 34
NMR spectra 36

Materials and Methods

Characterization: ^{1}H and ^{13}C NMR spectra were recorded with a JEOL AL-400 spectrometer (400 MHz for ^{1}H, 100 MHz for ^{13}C), a JEOL JNM-ECS400 (400 MHz for ^{1}H, 100 MHz for ^{13}C) or a JEOL JNM-ECA500II (500 MHz for ^{1}H, 126 MHz for ^{13}C) in $CDCl_3$. The chemical shifts in ^{1}H NMR spectra are reported in δ ppm using the residual proton of the solvent as an internal standard ($CHCl_3$ δ 7.26), and those in ^{13}C NMR spectra are reported using the solvent signal as an internal standard ($CDCl_3$ δ 77.16). Mass spectra were measured with a Thermo Fisher Scientific Exactive spectrometer with the ESI ionization method.

Synthesis: All reactions were performed with dry glassware and under a nitrogen atmosphere unless stated otherwise. Thin layer chromatography (TLC) was performed on glass plates coated with 0.25 mm thickness of silica gel 60F_{254} (Merck). Column chromatography was performed in self-packed columns using silica gel PSQ100B (Fuji Silysia Chemicals). Preparative Gel permeation Chromatography (GPC) was performed using LC-918 (Japan Analytical Industry) equipped with gel column (JAIGEL-2.5H and -3H) using $CHCl_3$ as eluent. All chemicals were purchased from commercial suppliers and used without further purification. Anhydrous THF was purchased from Kanto Chemicals and further purified by Glass Contour Solvent Systems.

UV-vis spectroscopy: The spectroscopic measurements were conducted under ambient conditions using solvents of spectroscopic grade. UV-vis absorption spectra were recorded using quartz cuvettes of 0.1 mm and 1 cm path length with a JASCO V-750 and a V-770 spectrophotometer equipped with a JASCO ETCR-762 cell holder for temperature control.

Photoluminescence spectroscopy: Fluorescence spectra were recorded using quartz cuvettes of 1 cm path length with a JASCO FP-8500 spectrometer.

Time-resolved photoluminescence spectroscopy: Prompt lifetimes (τ_p) were recorded using an EPLED picosecond pulsed LED source (365 nm) coupled with time-correlated single photon counting (TCSPC), while delayed lifetimes (τ_d) were recorded using a pulsed xenon microsecond flashlamp (Xe µF, λ_{ex} = 420 nm) coupled with multi-channel scaling (MCS). Lifetimes were analyzed by mono-exponential tail-fitting.

Photoluminescence quantum yield: Absolute photoluminescence quantum yields (PLQYs) were determined using an Edinburgh Instruments FS5 spectrofluorometer with the SC-30 Integrating Sphere Module with excitation at λ_{ex} = 420 nm.

Transmission electron microscopy: TEM was performed with a JEM-1400EM (JEOL) using an acceleration voltage of 80 kV. The samples (10 µL) were drop-casted on a carbon-coat copper grid (400 mesh) and the solvent was removed with a filter paper, followed by drying under reduced pressure. The measurements were conducted without additional staining.

Atomic force microscopy: AFM was performed under ambient conditions with a JSPM-5200V (JEOL) in NC mode. Silicon cantilevers (HQ:NSC35/Al BS, MikroMasch) with a resonance frequency of ~300 kHz and a force constant of ~16 Nm^{-1} were used, with silicon wafer as substrate.

Fourier-transform infrared spectroscopy: Fourier-transform infrared spectroscopy was performed on a JACSO FT/IR-4200 spectrometer.

Fluorescence microscopy: For confocal, photoluminescence and FLIM imaging a confocal laser scanning microscope (TCS SP8 FALCON gSTED; Leica) equipped with a pulsed white laser including Pulse Picker for FLIM (80, 40, 20, 10 MHz repetition rate), HyD detectors and HC PL APO CS2 100×/1.40 oil objective lens was used. The images were processed using Fiji. Photoluminescence and FLIM images were acquired under excitation at 420 nm and the emission was collected in the range of 450-550 nm. Data were collected using LAS X (Leica). The instrument response function (IRF) used for deconvolution was measured under the same microscope settings.

Dynamic light scattering: DLS was performed with a Zetasizer Nano particle analyser ZEN 3600 (Malvern) equipped with a 4 mW 632.8 nm laser using a 90° detector angle. Measurements were performed using quartz cuvettes of 1 cm path length.

Theoretical studies: All theoretical studies were carried out using the Gaussian 16 program.[1] Geometry optimizations were performed using the density functional theory (DFT) with the M052X as functional and 6-31G(d,p) as basis set. The solvation effects were included using the polarizable continuum model (PCM).[2]

Sample preparation: The compounds were dissolved in a stock solution at a high concentration (> 5.0×10^{-4} M) in chloroform. Afterwards the necessary amounts of the stock solution were transferred to a screw cap vial and the solvent was evaporated using a nitrogen stream followed by treatment *in vacuo* to complete dryness using a Schlenk line. To prepare the measurement solutions the necessary amount of solvent was added. For preparation in organic solvents the screw cap vial was heated using

a heatgun to dissolve the compound. For preparation in water the screw cap vial was sonicated at 31 kHz for no less than 10 minutes The measurement solutions were then transferred to the cuvettes immediately prior to measurement. For temperature dependent measurements the cuvettes were additionally sealed shut using a Teflon tape. Samples for co-assembly studies were prepared in the same fashion by preparing separated stock solutions of both compounds and transferring them to the same vial prior to evaporation.

Synthesis and Characterization

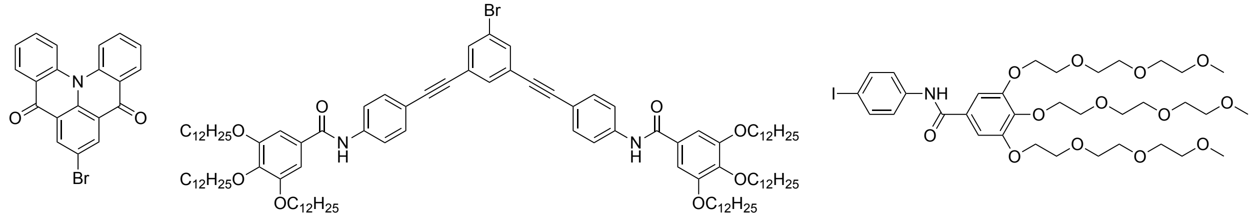

1 2 3

Scheme S1. Chemical structures of compounds **1**, **2**, and **3**.

Compounds **1**,[3] **2**[4] and **3**[5] were synthesized according to previously reported procedures and showed identical properties to those reported therein.

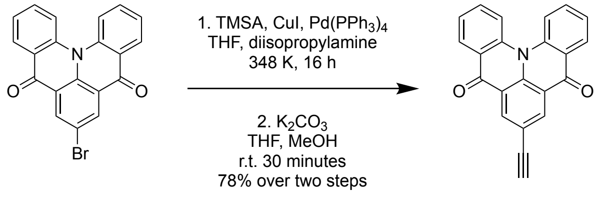

1 4

Scheme S2. Synthetic scheme for the preparation of compound **4**.

Compound **4**

Compound **1** (39 mg, 0.10 mmol, 1.0 eq.), CuI (3 mg, 0.02 mmol, 20 mol%) and $Pd(PPh_3)_4$ (30 mg, 0.03 mmol, 30 mol%) were dissolved in a mixture of dried tetrahydrofuran (THF, 35 mL) and diisopropylamine (15 ml) under a nitrogen atmosphere and stirred at room temperature for 30 min. Afterwards trimethylsilylacetylene (TMSA, 100 μL, 0.70 mmol, 7.0 eq.) was added dropwise and the reaction mixture was stirred at 348 K for 16 h. After cooling to room temperature, the solvent was removed under reduced pressure and the crude product was purified by column chromatography (SiO_2; hexane to DCM, R_f = 0.20 for 1/1 hexane/DCM). After confirming the full conversion of the starting material by ^{1}H NMR the crude product was suspended together with K_2CO_3 (140 mg, 1.0 mmol, 10.0 eq.) in a mixture of THF (35 mL) and methanol (15 ml) and stirred at room temperature for 30 min. Afterwards the solvent was removed under reduced pressure and the crude product was purified by column chromatography (SiO_2; hexane to DCM, R_f = 0.10 for 1/1 hexane/DCM) to give the target compound as an orange solid.

Yield: 25 mg, 0.08 mmol, 78%.

Mp: 200 °C (decomposition); ^{1}H NMR (400 MHz, 298 K, $CDCl_3$): δ 8.81 (s, 2H); 8.51–8.46 (m, 2H); 8.16–8.10 (m, 2H); 7.75–7.68 (m, 2H); 7.54–7.49 (m, 2H); 3.22 (s, 1H); ^{13}C NMR (100 MHz, 298 K, $CDCl_3$): δ 178.1, 139.8, 138.9. 136.1, 133.1, 128.1, 126.6, 125.7, 123.7, 120.5, 118.1, 81.6, 79.2; HRMS (ESI, positive): *m*/*z* calcd. For $C_{22}H_{12}NO_2$: 322.0868 ([*M*+H]$^+$); found: 322.0862.

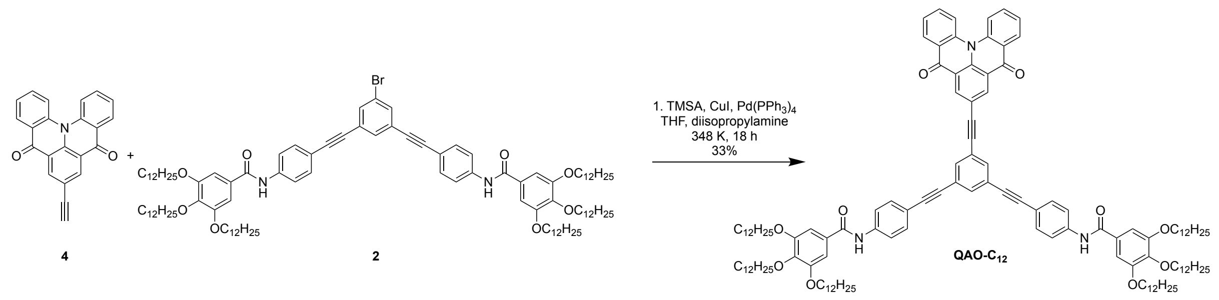

Scheme S3. Synthetic scheme for the preparation of **QAO-C$_{12}$**.

Compound **QAO-C$_{12}$**

Compound **4** (25 mg, 0.078 mmol, 1.0 eq.), compound **2** (178 mg, 0.105 mmol, 1.35 eq.), CuI (1 mg, 0.005 mmol, 7 mol%) and Pd(PPh$_3$)$_4$ (10 mg, 0.009 mmol, 11 mol%) were dissolved in a mixture of dried THF (60 mL) and diisopropylamine (20 ml) under a nitrogen atmosphere and stirred at room temperature for 30 min. Afterwards the reaction mixture was stirred at 348 K for 18 h. After cooling to room temperature, the solvent was removed under reduced pressure and the crude product was purified by column chromatography (SiO$_2$; DCM to 19:1 DCM/ethylacetate, R_f = 0.44 (DCM)). The product was further purified by preparative GPC using chloroform as eluent to give the target compound as a bright yellow solid.

Yield: 50 mg, 0.026 mmol, 33%.

Mp: 185-186 °C; ^{1}H NMR: (400 MHz, 298 K, CDCl$_3$): δ = 8.84 (s, 2H); 8.51–8.47 (m, 2H); 8.16–8.11 (m, 2H); 7.86 (s, 2H); 7.75–7.70 (m, 2H); 7.69–7.66 (m, 6H); 7.65–7.64 (m, 1H); 7.58–7.54 (m, 4H); 7.53–7.48 (m, 2H); 7.05 (s, 4H); 4.06–3.99 (m, 12H); 1.86–1.71 (m, 12H); 1.52–1.42 (m, 12H); 1.38–1.24 (m, 96H); 0.90–0.85 (m, 18H); ^{13}C NMR (100 MHz, 298 K, CDCl$_3$): δ 178.0, 165.9, 153.3, 141.6, 139.7, 138.6, 138.5, 135.5, 134.4, 134.0, 133.1, 132.7, 129.8, 128.1, 126.5, 125.6, 124.3, 123.6, 123.4, 120.5, 120.0, 118.8, 118.6, 105.9, 90.7, 89.8, 88.4, 87.7, 73.7, 69.5, 32.1, 30.5, 29.91, 29.86, 29.8, 29.7, 29.58, 20.55, 29.52, 29.49, 26.2, 22.8, 14.3; HRMS (ESI, negative): m/z calcd. for $C_{130}H_{177}N_3O_{10}Cl$: 1975.3122 ([$M$+Cl]$^-$); found: 1975.3123.

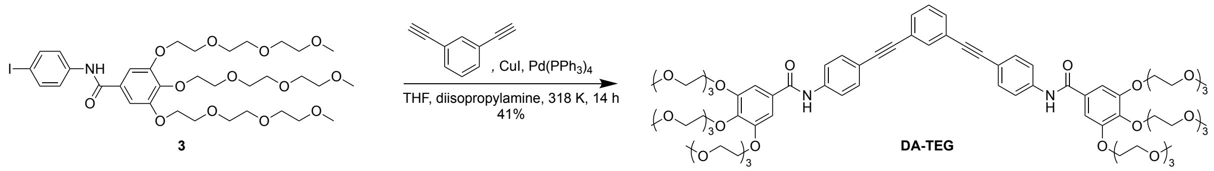

Scheme S4. Synthetic scheme for the preparation of **DA-TEG**.

Compound **DA-TEG**

Compound **3** (356 mg, 0.44 mmol, 2.2 eq.), CuI (4 mg, 0.02 mmol, 10 mol%) and $Pd(PPh_3)_4$ (40 mg, 0.035 mmol, 17 mol%) were dissolved in a mixture of dried THF (40 mL) and diisopropylamine (20 ml) under a nitrogen atmosphere and stirred at room temperature for 110 min. Afterwards, 1,3-diethynylbenzene (28.4 μL, 25 mg, 0.20 mmol, 1.0 eq) was added and the reaction mixture was stirred at 318 K for 14 h. After cooling to room temperature, the solvent was removed under reduced pressure and the crude product was purified by column chromatography (SiO_2; DCM to 19:1 DCM/MeOH, R_f = 0.41 for 19/1 DCM/MeOH). The product was further purified by preparative GPC using chloroform as eluent to give the target compound as a brown–yellow highly viscous oil.

Yield: 122 mg, 0.082 mmol, 41%.

^{1}H NMR: (400 MHz, 298 K, $CDCl_3$): δ 8.88 (s, 2H); 7.75–7.70 (m, 4H); 7.68–7.67 (m, 1H); 7.51–7.47 (m, 4H); 7.46–7.43 (m, 2H); 7.33–7.28 (m, 1H); 7.20 (s, 4H); 4.20–4.12 (m, 12H); 3.81–3.73 (m, 12H); 3.79–3.66 (m, 12H); 3.65–3.58 (m, 24H); 3.53–3.47 (m, 12H); 3.34 (s, 6H); 3.29 (s, 12H); ^{13}C NMR (100 MHz, 298 K, $CDCl_3$): δ 165.8, 152.5, 141.9, 138.9, 134.5, 132.4, 131.1, 129.9, 128.6, 123.8, 120.3, 118.5, 107.8, 90.0, 88.3, 72.4, 72.0, 71.9, 70.72, 70.69, 70.65, 70.61, 70.56, 70.5, 69.8, 69.1, 59.1, 59.0; HRMS (ESI, negative): *m/z* calcd. for $C_{78}H_{107}N_2O_{26}$: 1487.7112 ([*M*–H]$^-$); found: 1487.7119.

Additional Spectroscopy

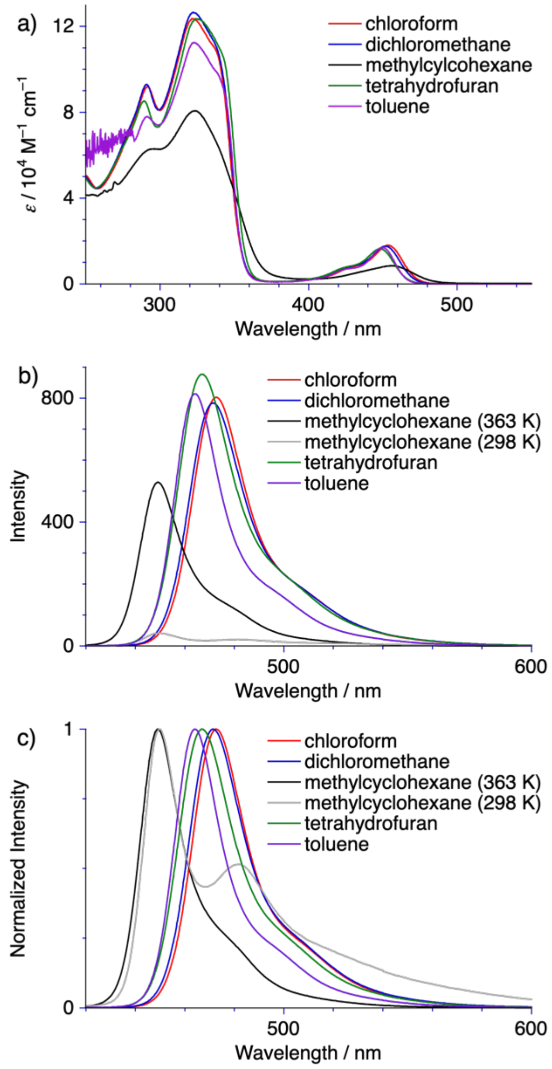

Figure S1. a) Solvent-dependent UV-vis absorption spectra of **QAO-C$_{12}$** at T = 298 K and c = 1.0 × 10^{-5} M. b,c) Solvent-dependent photoluminescence and normalized photoluminescence spectra of **QAO-C$_{12}$** (λ_{ex} = 310 nm) at T = 298 K and c = 1.0 × 10^{-5} M and the spectra at T = 363 K in MCH.

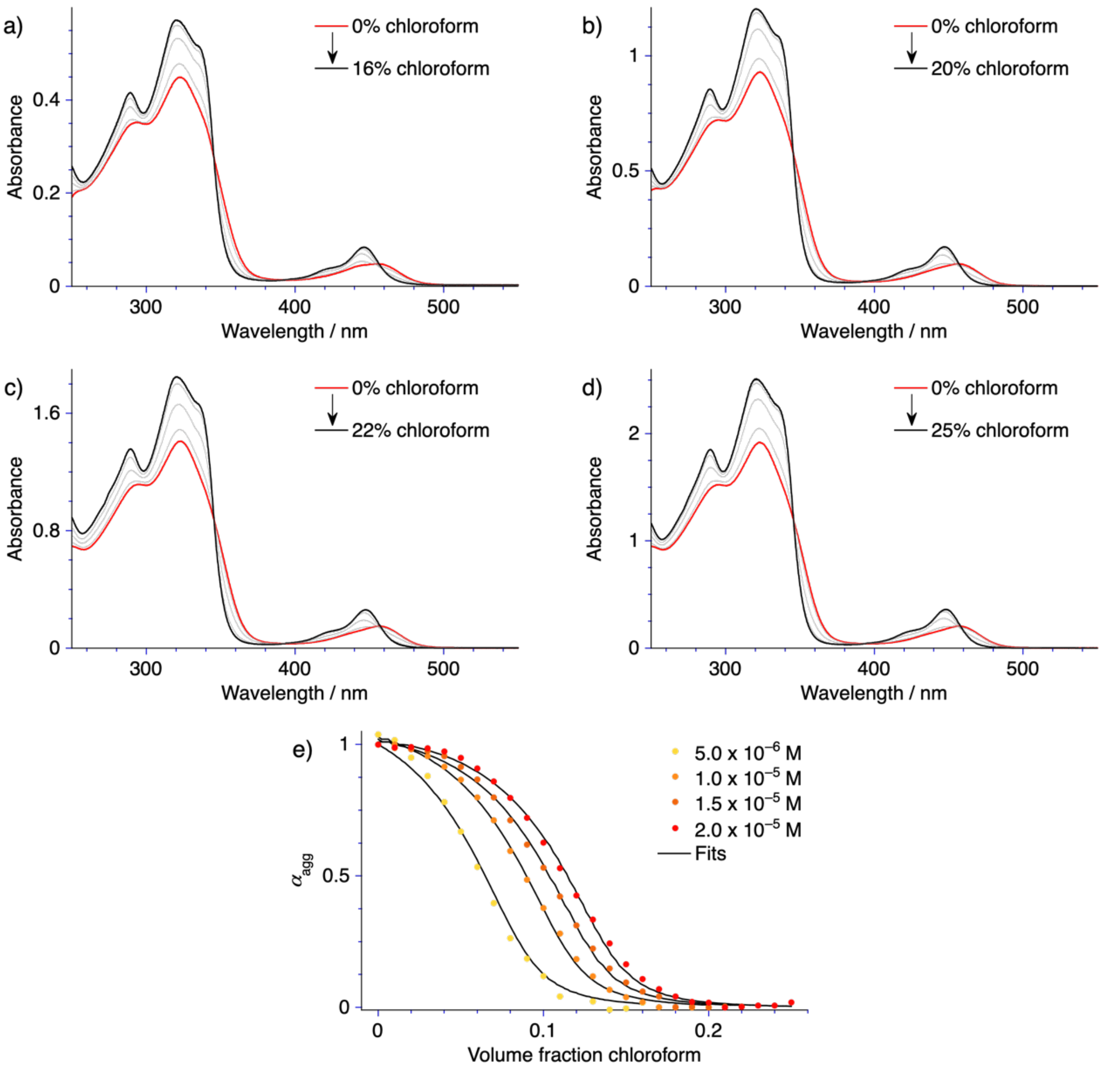

Figure S2. a-d) Solvent-dependent UV-vis absorption spectra of **QAO-C$_{12}$** using MCH as self-assembly inducing and chloroform as denaturing agent at T = 298 K and c = 5.0 × 10^{-6} M (a); c = 1.0 × 10^{-5} M (b); c = 1.5 × 10^{-5} M (c) and c = 2.0 × 10^{-5} M (d). e) Aggregation parameter (α_{agg}) calculated based on the absorbance at λ_{abs} = 465 nm plotted against the volume fraction of chloroform with cooperative fits derived from the solvent-dependent model by Meijer and co-workers using a global fitting approach.[6]

Table S1. Thermodynamic parameters derived from fitting the denaturation curves in Fig.S2 using MCH as self-assembly inducing and chloroform as denaturing agent at T = 298 K to the solvent-dependent model using a global fitting approach.[6]

ΔG^0 / kJ·mol^{-1}	ΔG^0 (SD) / kJ·mol^{-1}	m / kJ·mol^{-1}	m (SD) / kJ·mol^{-1}	σ / 10^{-2}	σ (SD) / 10^{-3}
–35.2	1.5	64.7	1.7	4.6	6.3

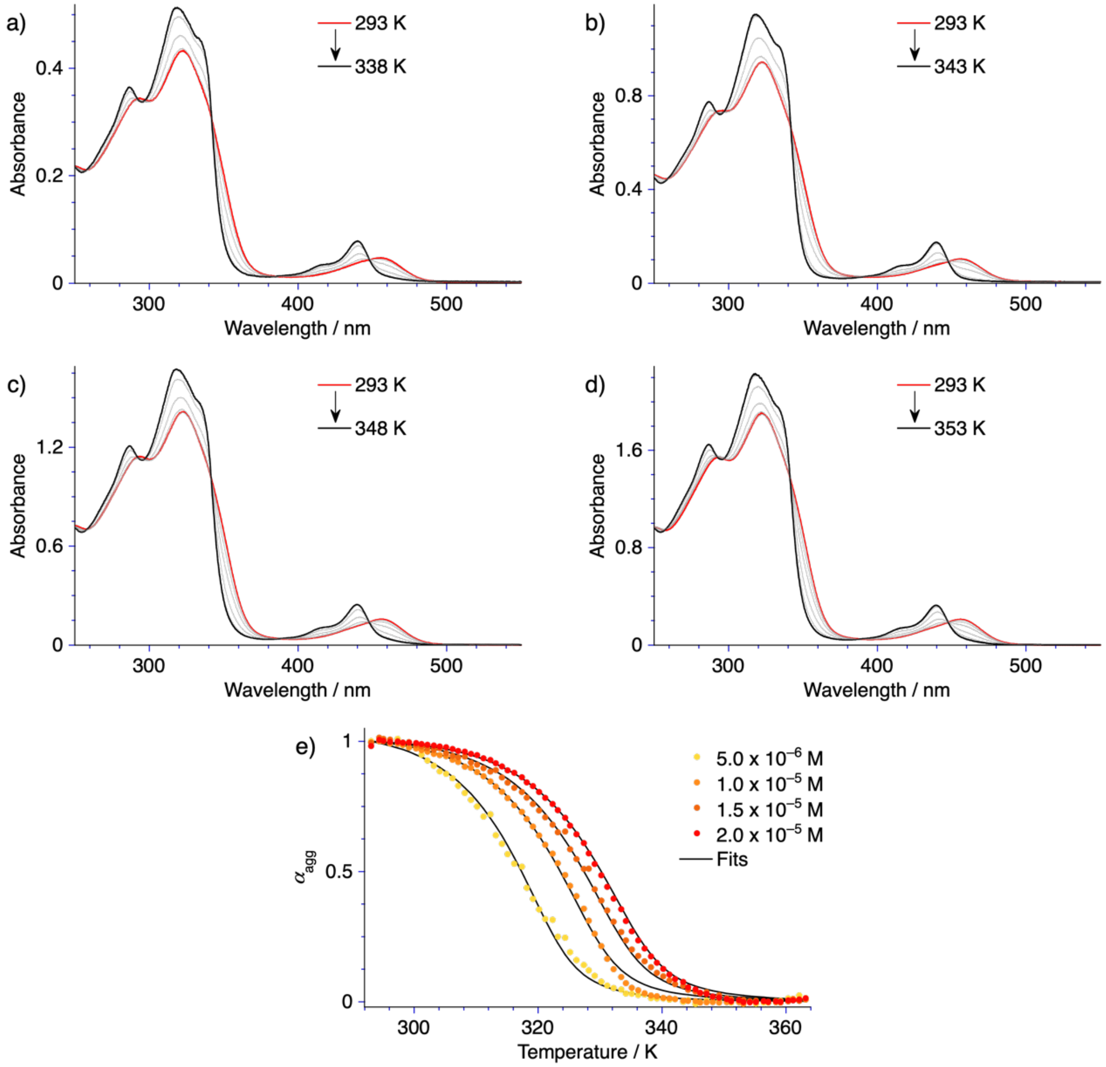

Figure S3. a-d) Temperature-dependent UV-vis absorption spectra of **QAO-C**$_{12}$ in MCH between T = 293 K and T = 353 K using a heating rate of 1 K/min and a data interval of 1 K at c = 5.0 × 10^{-6} M (a); c = 1.0 × 10^{-5} M (b); c = 1.5 × 10^{-5} M (c) and c = 2.0 × 10^{-5} M (d). e) Aggregation parameter (α_{agg}) calculated based on the absorbance at λ_{abs} = 465 nm plotted against the temperature with cooperative fits derived from the nucleation-elongation model using a global fitting approach.[7]

The elongation enthalpy (ΔH_e^0), nucleation penalty (ΔH_n^0), entropy (ΔS^0) and elongation temperature (T_e) were obtained directly from the fitting procedure, while the elongation (K_e) and nucleation (K_n) equilibrium constants, and degree of cooperativity (σ) were calculated using equation 1–3:[7]

$$K_n = e\left(\frac{-\left((\Delta H_e - \Delta H_n) - T_e \Delta S\right)}{RT_e}\right) \;(1)$$

$$K_e = e\left(\frac{-(\Delta H_e - T_e \Delta S)}{RT_e}\right) \;(2)$$

$$\sigma = \frac{K_n}{K_e} \;(3)$$

Table S2. Thermodynamic parameters derived from fitting the heating curves in Fig.S3 to the nucleation-elongation model using a global fitting approach.[7]

c / 10^{-5} M	ΔH_e (SD) / kJ·mol^{-1}	ΔH_n (SD) / kJ·mol^{-1}	ΔS (SD) / kJ·mol^{-1}·K^{-1}	$\Delta G^{0\,a}$ / kJ·mol^{-1}	T_e (SD) / K	K_e / 10^5 M^{-1}	K_n / 10^3 M^{-1}	σ / 10^{-2}
0.5	–92.8 (1.0)	–8.6 (0.2)	–0.187 (0.003)	–36.9	321.3 (0.1)	20.0	8.1	4.1
1.0					327.9 (0.1)	10.0	4.3	4.3
1.5					331.8 (0.2)	6.7	3.0	4.5
2.0					334.7 (0.2)	5.0	2.3	4.6

[a] The Gibbs free energy was determined for a standard temperature of T = 298 K.

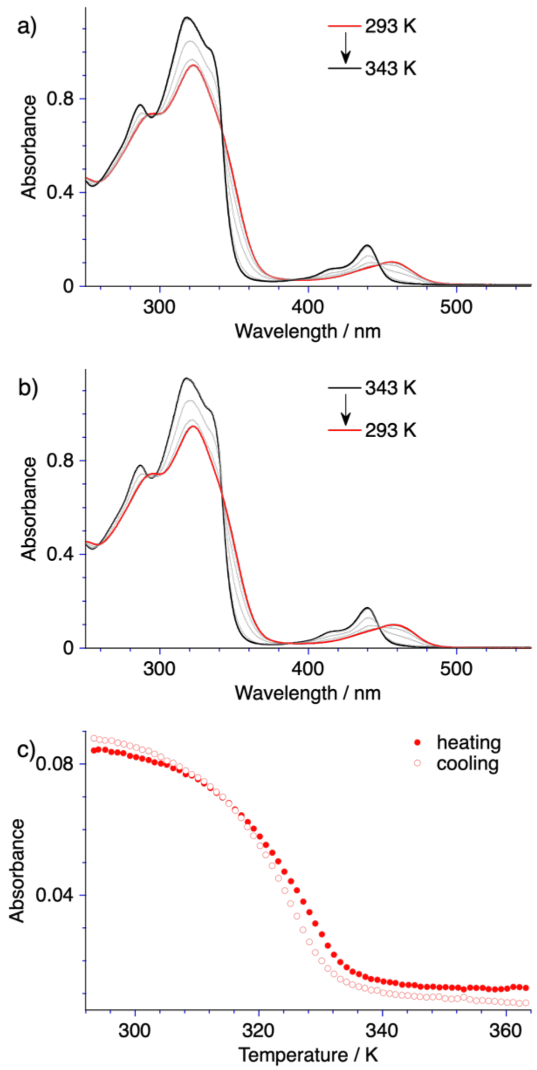

Figure S4. a-b) Temperature-dependent heating (a) and cooling (b) UV-vis absorption spectra of **QAO-C$_{12}$** in MCH between T = 293 K and T = 353 K using a heating rate of 1 K/min and a data interval of 1 K at c = 1.0 × 10^{-5} M. c) Absorbance at λ_{abs} = 465 nm plotted against the temperature for heating and cooling experiments in (a) and (b).

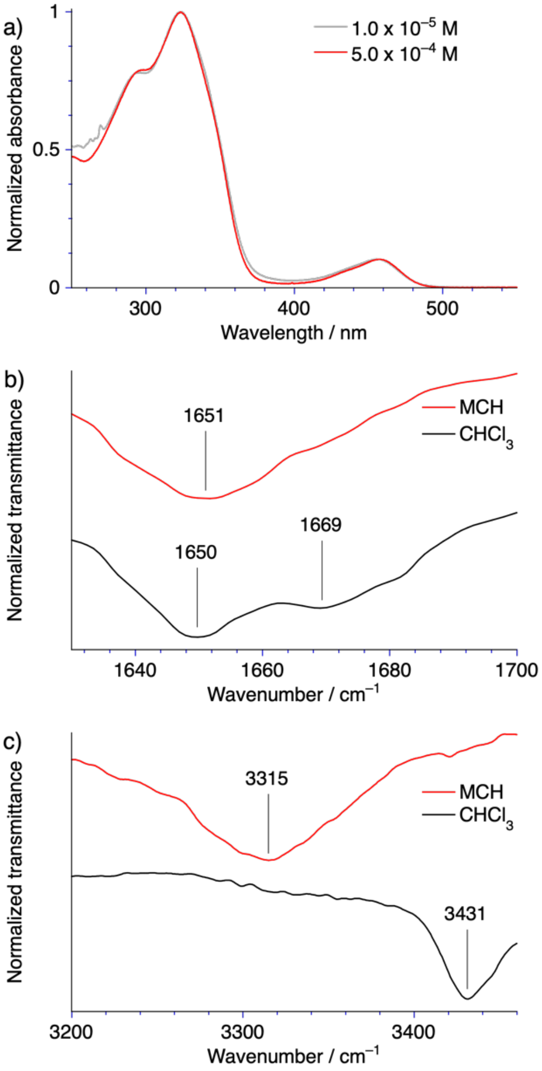

Figure S5. a) Concentration-dependent UV-vis absorption spectra of **QAO-C12** in MCH at T = 298 K. b,c) Normalized solvent-dependent FT-IR spectra of **QAO-C12** highlighting the C=O stretching frequency (b) and the N-H stretching frequency (c) respectively, recorded at T = 298 K and c = 5.0 × 10^{-4} M.

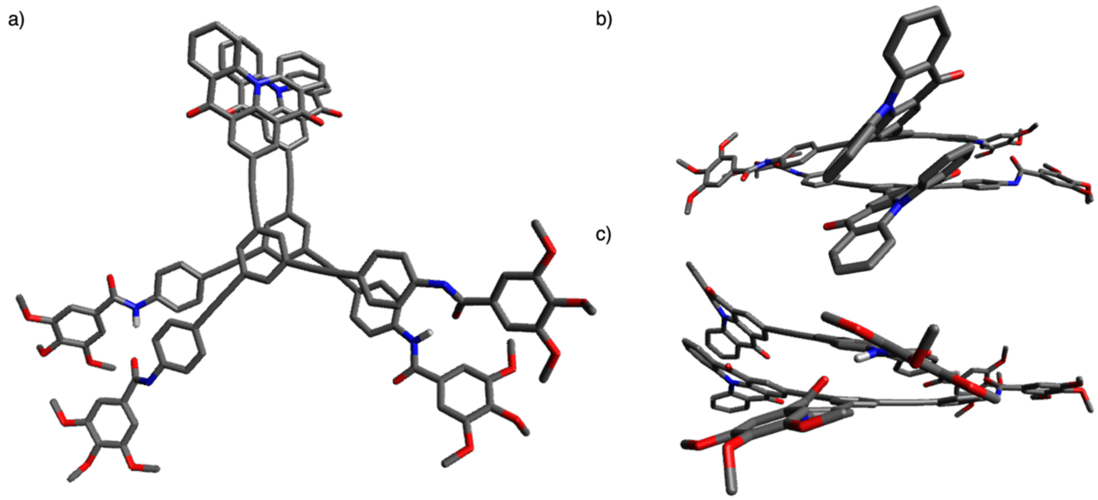

Figure S6. Optimized dimer structure of **QAO-Me** from the top (a), as well as along the alkyne bond connecting the central benzene moiety to the QAO (b) and the amide (c) groups. Calculations were performed at the M052X/6-31G(d,p) level of theory with methylcyclohexane as solvent using the polarizable continuum model. Hydrogens that are not involved in hydrogen bonding have been omitted for clarity.

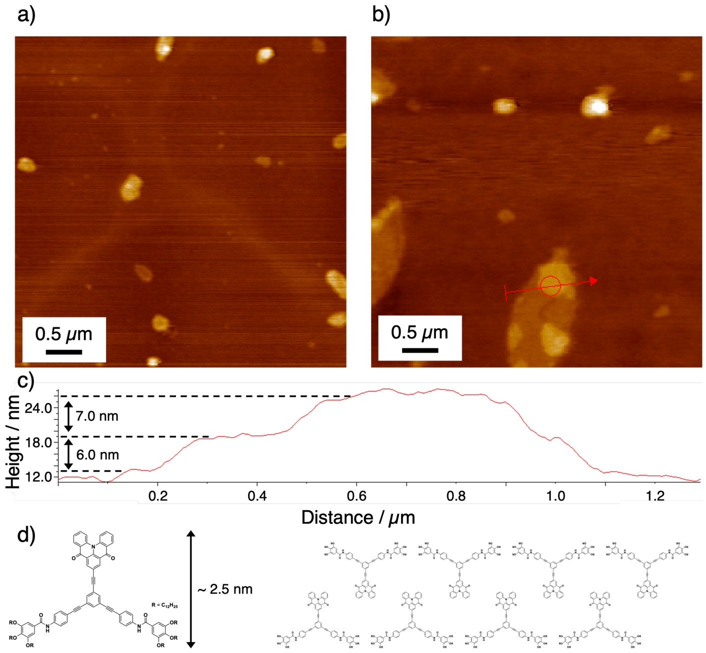

Figure S7. a,b) AFM micrographs of **QAO-C12** obtained from dropcasting (V = 10 μL) a MCH solution at c = 5.0 × 10^{-5} M (a) and c = 2.0 × 10^{-5} M (b). Scale bars correspond to 0.5 μm. c) Height profile along the red line in (b). d) Molecular representation of **QAO-C12** with proposed packing model leading to efficient shielding of the polar carbonyl groups.

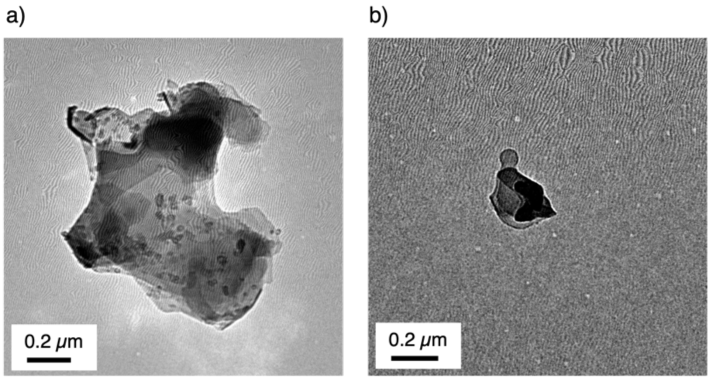

Figure S8. a,b) TEM micrographs of **QAO-C$_{12}$** obtained from dropcasting (V = 10 μL) a MCH solution at c = 5.0 × 10^{-5} M (a) and 2.0 × 10^{-5} M. Scale bars correspond to 0.2 μm.

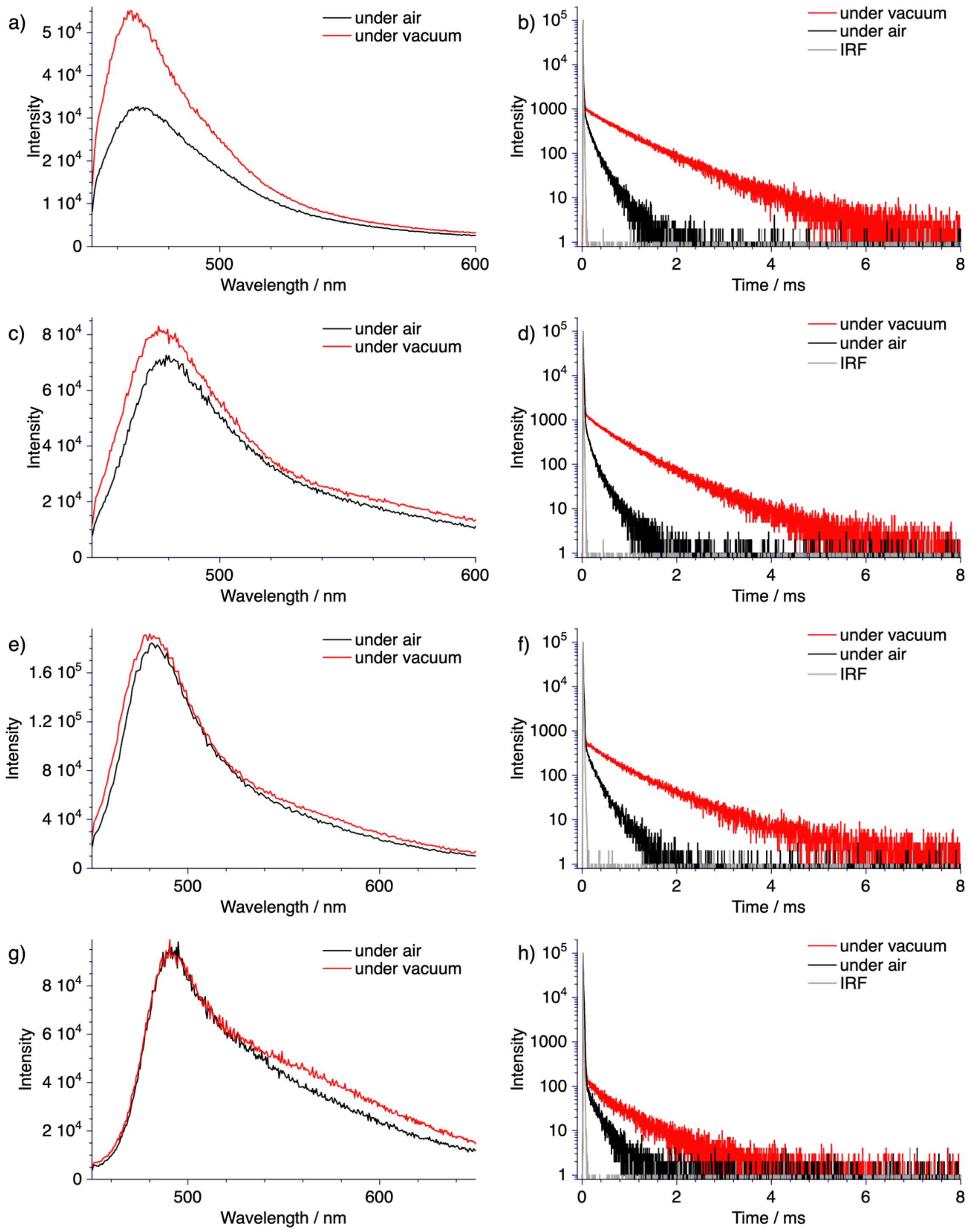

Figure S9. a,c,e,g) Photoluminescence spectra (λ_{ex} = 420 nm) of a 1 wt.% (a), a 5 wt.% (c), a 10 wt.% (e) and a 50 wt.% (g) PMMA film of **QAO-C$_{12}$**. b,d,f,h) Photoluminescence decays of the thin films shown in (a,c,e,g) under vacuum and aerated conditions recorded at λ_{em} = 467 nm (b), λ_{em} = 476 nm (d), λ_{em} = 480 nm (f) and λ_{em} = 495 nm (h). IRF = instrument response function.

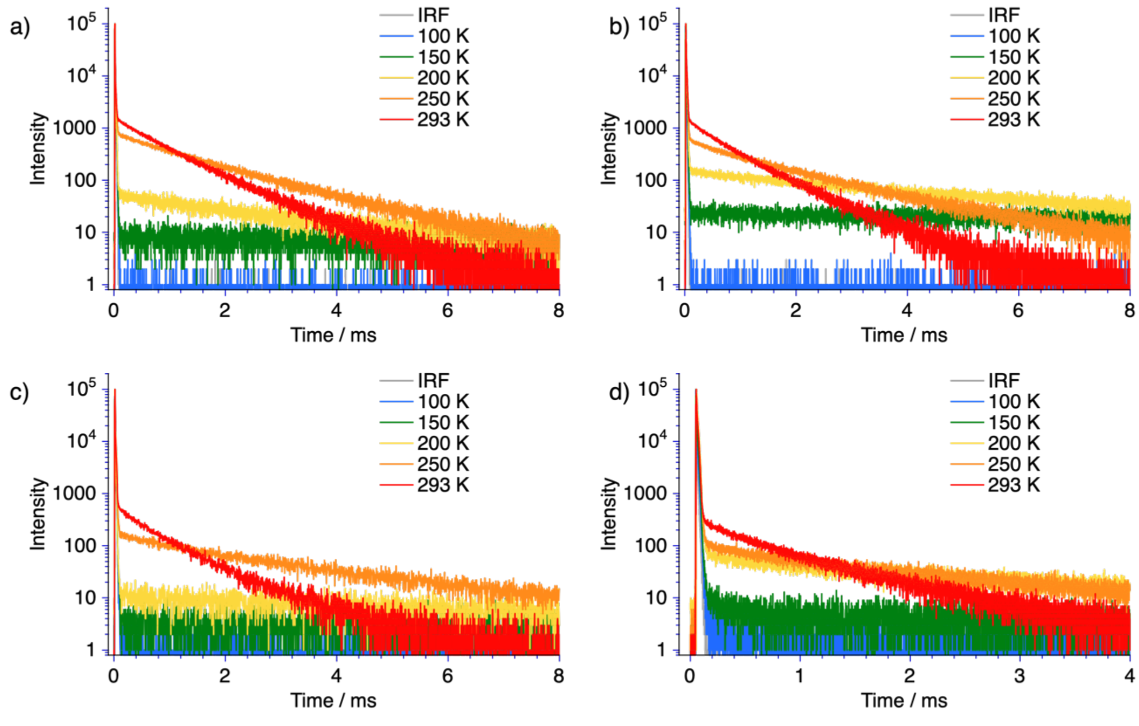

Figure S10. Temperature-dependent photoluminescence decays of a 1 wt.% (a), a 5 wt.% (b), a 10 wt.% (c) and a 50 wt.% doped PMMA film (d) of **QAO-C$_{12}$** recorded at λ_{em} = 467 nm (a), λ_{em} = 476 nm (b), λ_{em} = 480 nm (c) and λ_{em} = 495 nm (d). IRF = instrument response function.

The prompt lifetimes ($\tau_{PF,air}$) and PLQYs of the prompt fluorescence ($\Phi_{PL,air}$) were obtained experimentally with doped PMMA films of **QAO-C$_{12}$** under aerated condition. The intensities of the prompt fluorescence ($I_{PF,air}$) and the total emission (including both prompt and delayed components, $I_{tot,vac}$) were determined by integrating the areas under the emission curves measured under aerated and vacuum conditions, respectively. Thus, PLQYs of the total emission (Φ_{PLQY}) were calculated using equation 5. The rate constants of radiative (k_r) and non-radiative (k_{nr}) decay processes can therefore be calculated using equations 4-7.[8]

$$k_{PF} = \frac{1}{\tau_{PF,air}} \quad (4)$$

$$\Phi_{PLQY} = \Phi_{PF,air}\frac{I_{tot,vac}}{I_{PF,air}} \quad (5)$$

$$k_r = k_{PF}\Phi_{PF,air} \quad (6)$$

$$k_{nr} = k_{PF}\frac{\Phi_{PF,air}}{\Phi_{PLQY}}(1-\Phi_{PLQY}) \quad (7)$$

Table S3. Photophysical properties of doped PMMA films of **QAO-C$_{12}$** determined at the photoluminescence maximum (λ_{em}). FWHM = full width at half maximum, PL = photoluminescence, PF = prompt fluorescence, DF = delayed fluorescence, r = radiative decay, nr = non-radiative decay.

Doping / wt.%	λ_{em} (FWHM) / nm	$\Phi_{PL,air}$ / %	$\tau_{PF,air}$ ($\tau_{PF,vac}$) / ns	$\tau_{DF,air}$ ($\tau_{DF,vac}$) / µs	k_r / 10^6 s^{-1}	k_{nr} / 10^7 s^{-1}
1	467 (39)	5.5	4.55 (4.80)	195 (830)	1.21	1.38
5	476 (47)	5.1	2.43 (2.73)	195 (752)	2.10	3.35
10	480 (47)	3.6	3.19 (3.33)	208 (655)	1.13	2.74
50	495 (62)	1.5	2.37 (2.75)	204 (524)	0.63	3.77

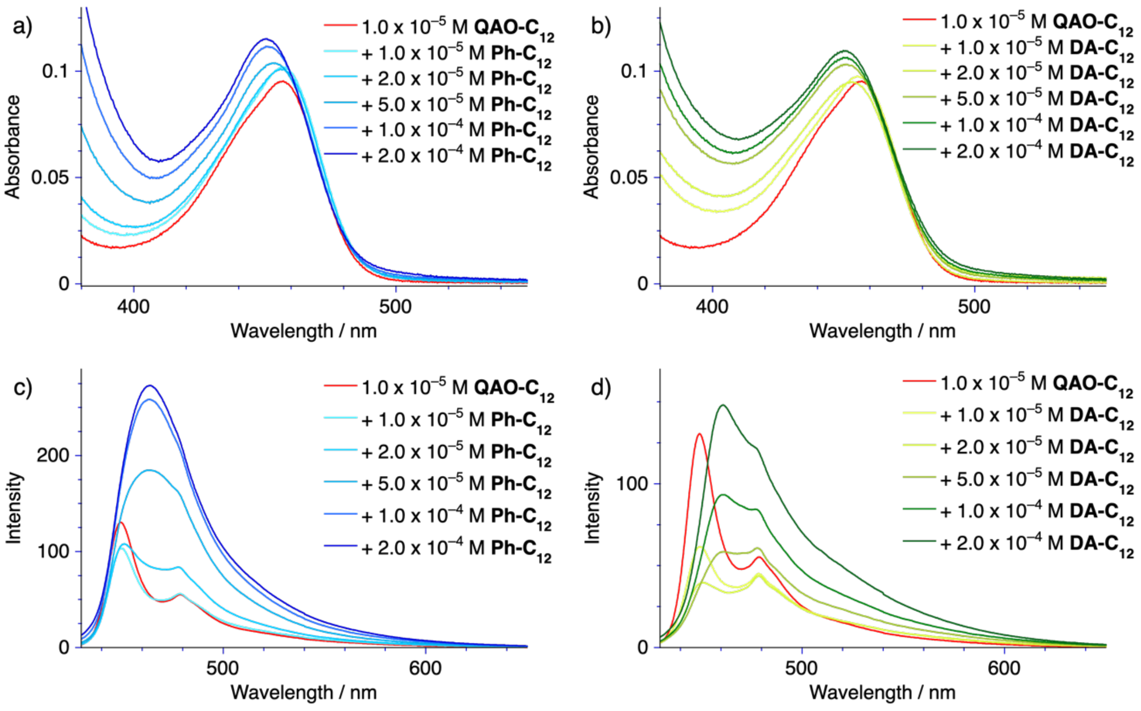

Figure S11. a-d) UV-vis (a,b) and photoluminescence (c,d, λ_{ex} = 420 nm) spectra of **QAO-C$_{12}$** in MCH at T = 298 K and c = 1.0 × 10^{-5} M in the presence of increasing amounts of **Ph-C$_{12}$** (a,c) and **DA-C$_{12}$** (b,d).

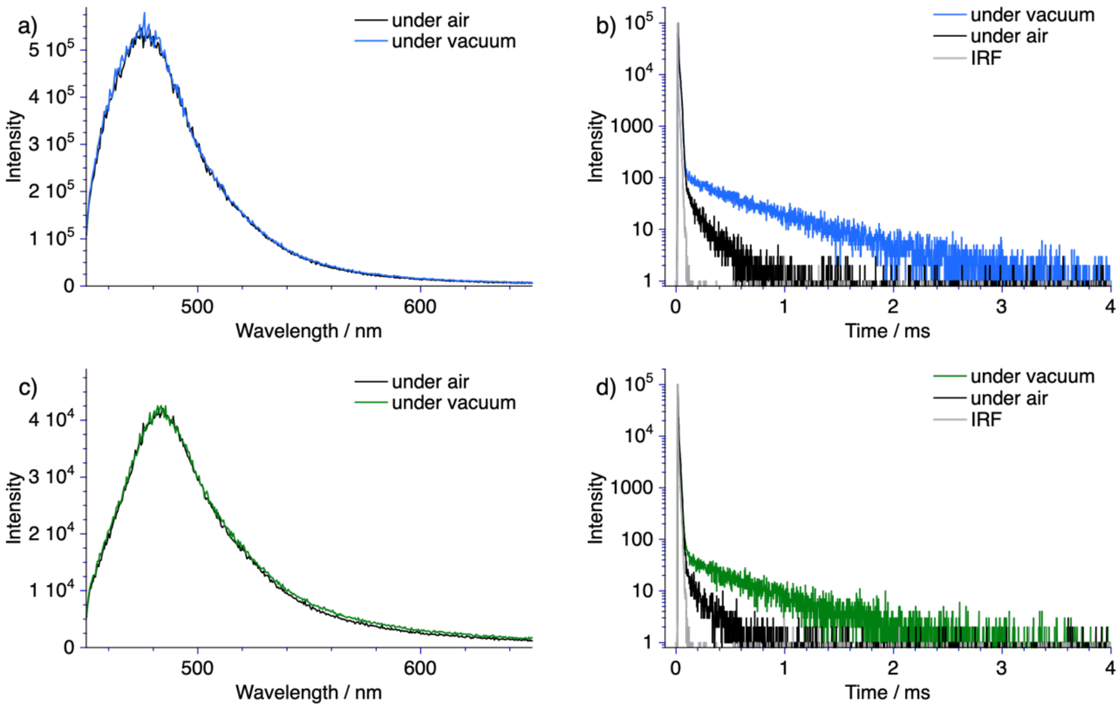

Figure S12. a,c) Photoluminescence decays (λ_{ex} = 420 nm) of **QAO-C_{12}.Ph-C_{12}** (λ_{em} = 475 nm) and **QAO-C_{12}.DA-C_{12}** (λ_{em} = 484 nm) copolymers in a 50 wt.% doped PMMA film (5% **QAO-C_{12}**, 45% modulator) under vacuum. b,d) Photoluminescence decays of the thin films shown in (a and c) under vacuum and aerated conditions recorded at λ_{em} = 475 nm (b) and λ_{em} = 484 nm (d). IRF = instrument response function.

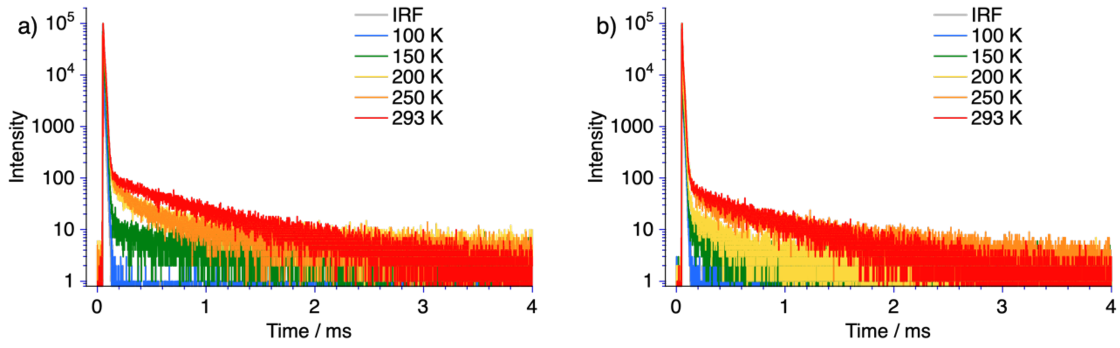

Figure S13. Temperature dependent photoluminescence decays of **QAO-C_{12}.Ph-C_{12}** (a, λ_{em} = 475 nm) and **QAO-C_{12}.DA-C_{12}** (b, λ_{em} = 484 nm) copolymers in a 50 wt.% doped PMMA film (5% **QAO-C_{12}**, 45% modulator) under vacuum. IRF = instrument response function.

Table S4. Photophysical properties of **QAO-C12.Ph-C12** (λ_{em} = 475 nm) and **QAO-C12.DA-C12** (λ_{em} = 484 nm) copolymers in a 50 wt.% doped PMMA film (5% **QAO-C12**, 45% modulator) determined at the photoluminescence maximum (λ_{em}). FWHM = full width at half maximum, PL = photoluminescence, PF = prompt fluorescence, DF = delayed fluorescence.

Copolymer	λ_{em} (FWHM) / nm	$\Phi_{PL,air}$ / %	$\tau_{PF,air}$ ($\tau_{PF,vac}$) / ns	$\tau_{DF,air}$ ($\tau_{DF,vac}$) / µs
**QAO-C12.Ph-C12**	475 (42)	4.9	2.44 (2.49)	126 (545)
**QAO-C12.DA-C12**	484 (45)	2.9	2.65 (2.69)	102 (464)

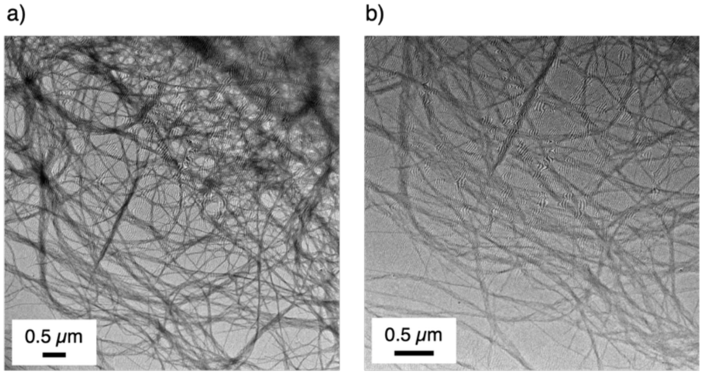

Figure S14. a,b) TEM micrographs obtained from dropcasting (V = 10 μL) solutions of **QAO-C12** at c = 5.0 × 10^{-5} M and **DA-C12** at c = 5.0 × 10^{-4} M in MCH at T = 298 K after aging the solutions for 5 days. Scale bars correspond to 0.5 μm. The observed fibrillar assemblies correspond to self-sorted homopolymers of **DA-C12**.[9]

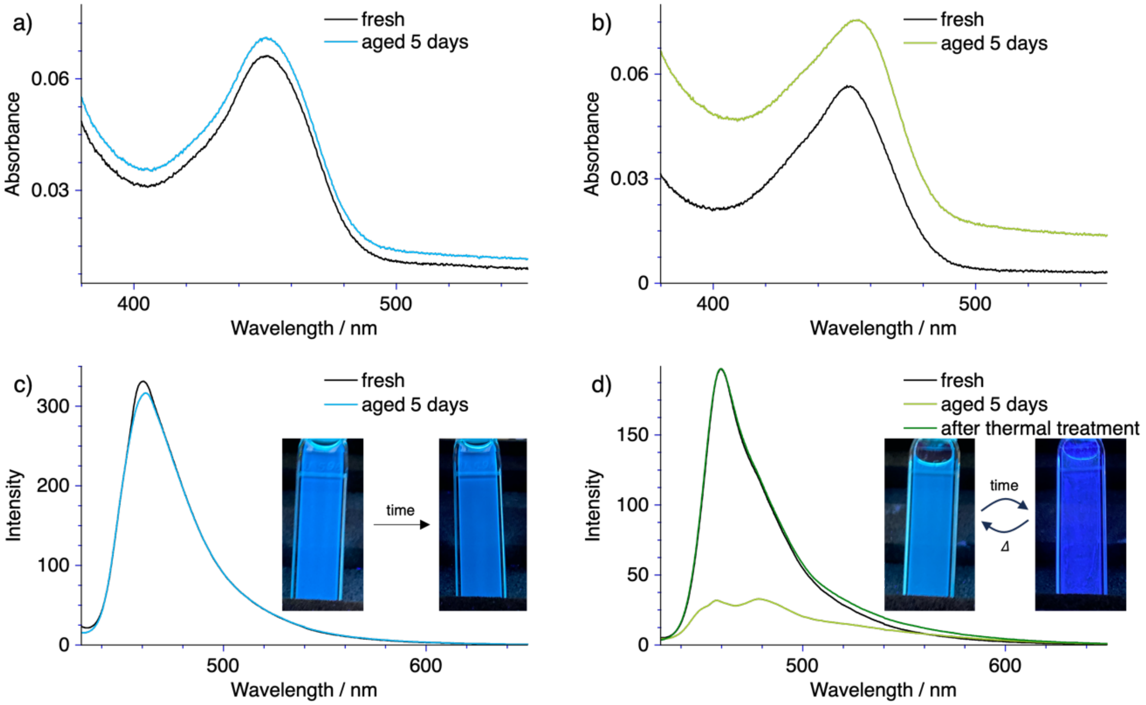

Figure S15. a-d) Time-dependent UV-vis (a,b) and photoluminescence (c,d, λ_{ex} = 420 nm) spectra of **QAO-C12** in MCH at T = 298 K and c = 5.0 × 10^{-5} M in the presence of **Ph-C12** (a,c) and **DA-C12** (b,d) at c = 5.0 × 10^{-4} M. Insets: photographs of the corresponding cuvettes under UV-light irradiation (λ_{ex} = 365 nm).

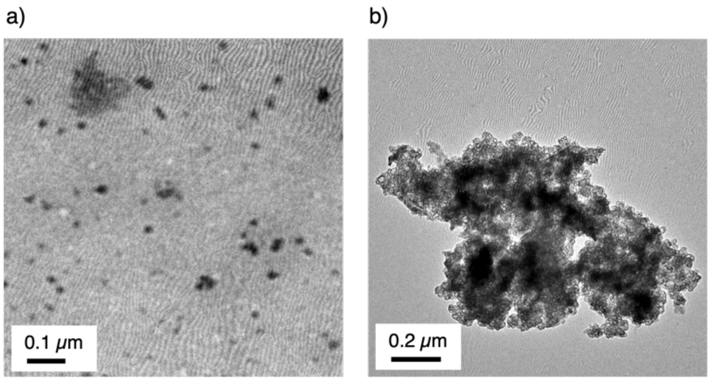

Figure S16. a,b) TEM micrographs of **QAO-C12** obtained from dropcasting (V = 10 μL) a MCH solution at $c = 2.0 \times 10^{-5}$ M in the presence of **Ph-C12** (a) and **DA-C12** (b) at $c = 5.0 \times 10^{-4}$ M. Scale bars correspond to 0.1 (a) and 0.2 μm (b).

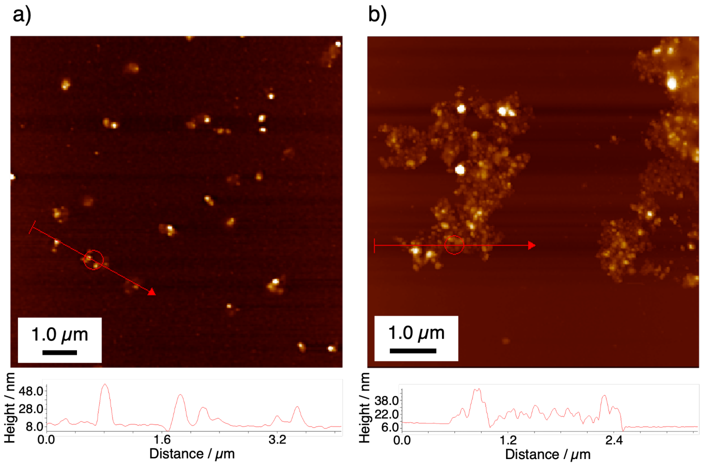

Figure S17. a,b) AFM micrographs of **QAO-C12** obtained from dropcasting (V = 10 μL) a MCH solution at $c = 2.0 \times 10^{-5}$ M in the presence of **Ph-C12** (a) and **DA-C12** (b) at $c = 5.0 \times 10^{-4}$ M. Scale bars correspond to 1.0 μm. Height profiles along the red lines are shown below the respective micrograph.

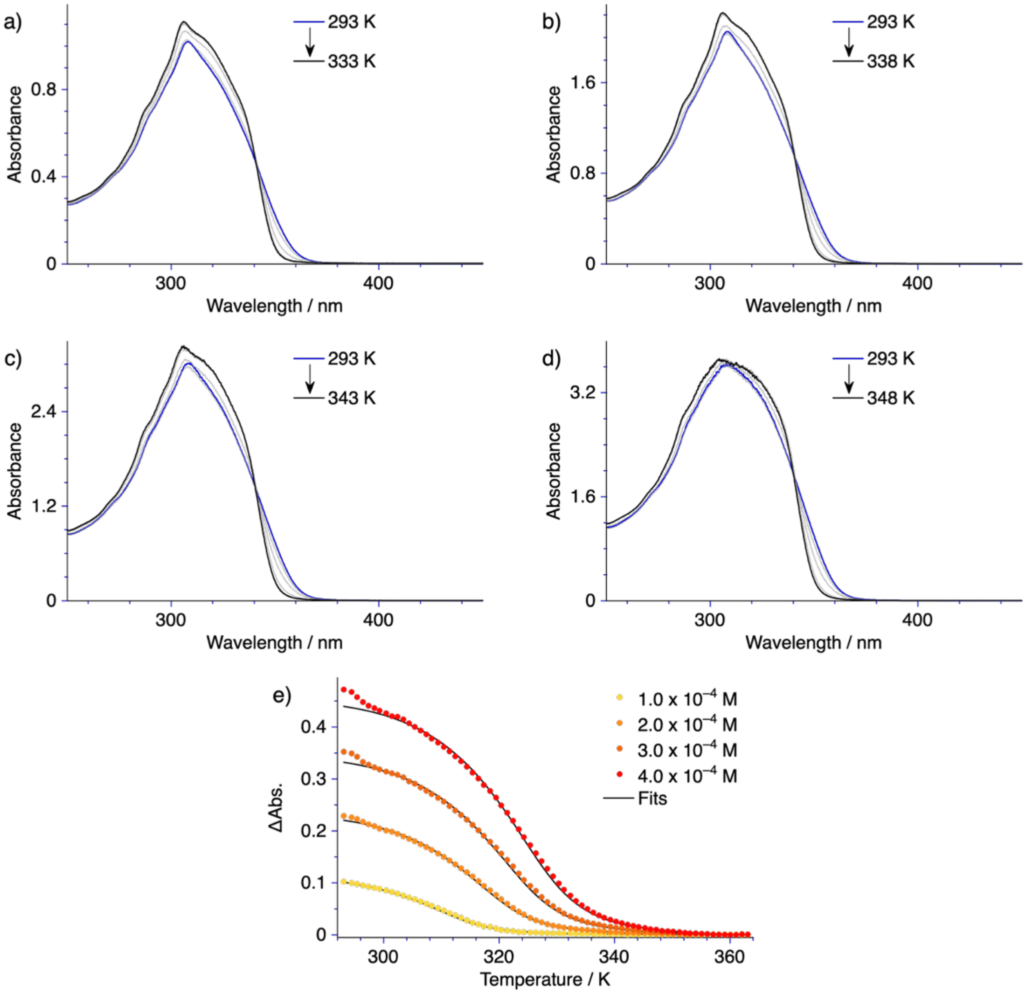

Figure S18. a-d) Temperature-dependent UV-vis absorption spectra of **Ph-C12** in MCH between T = 293 K and T = 348 K using a heating rate of 1 K/min and a data interval of 1 K at c = 1.0 × 10^{-4} M (a); c = 2.0 × 10^{-4} M (b); c = 3.0 × 10^{-4} M (c) and c = 4.0 × 10^{-4} M (d). e) Changes of the absorbance at λ_{abs} = 355 nm plotted against the temperature with cooperative fits derived from the nucleation-elongation model using a global fitting approach.[7]

Table S5: Thermodynamic parameters derived from fitting the heating curves in Fig.S16 to the nucleation-elongation model using a global fitting approach.[7]

c / 10^{-4} M	ΔH_e (SD) / kJ·mol^{-1}	ΔH_n (SD) / kJ·mol^{-1}	ΔS (SD) / kJ·mol^{-1}·K^{-1}	$\Delta G^{0\,a}$ / kJ·mol^{-1}	T_e (SD) / K	K_e / 10^4 M^{-1}	K_n / 10^2 M^{-1}	σ / 10^{-2}
1.0	–80.2 (0.8)	–7.6 (0.1)	–0.181 (0.003)	–26.4	311.8 (0.1)	10.0	5.3	5.3
2.0					319.0 (0.1)	5.0	2.8	5.7
3.0					323.3 (0.2)	3.3	2.0	5.9
4.0					326.5 (0.2)	2.5	1.5	6.1

[a] The Gibbs free energy was determined for a standard temperature of T = 298 K.

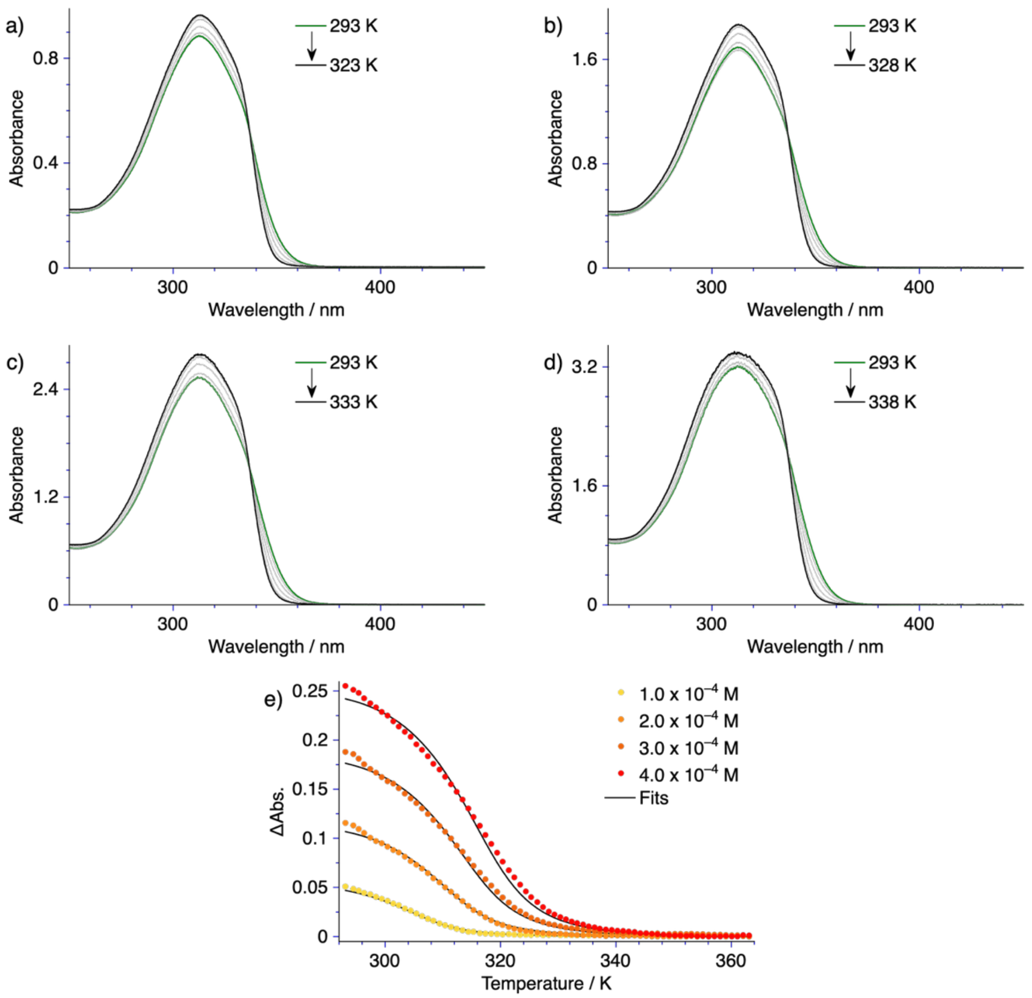

Figure S19. *a*-d) Temperature-dependent UV-vis absorption spectra of **DA-C12** in MCH between T = 293 K and T = 348 K using a heating rate of 1 K/min and a data interval of 1 K at $c = 1.0 \times 10^{-4}$ M (a); $c = 2.0 \times 10^{-4}$ M (b); $c = 3.0 \times 10^{-4}$ M (c) and $c = 4.0 \times 10^{-4}$ M (d). e) Changes of the absorbance at λ_{abs} = 355 nm plotted against the temperature with cooperative fits derived from the nucleation-elongation model using a global fitting approach.[7]

Table S6. Thermodynamic parameters derived from fitting the heating curves in Fig.S17 to the nucleation-elongation model using a global fitting approach.[7]

c / 10^{-4} M	ΔH_e (SD) / kJ·mol^{-1}	ΔH_n (SD) / kJ·mol^{-1}	ΔS (SD) / kJ·mol^{-1}·K^{-1}	$\Delta G^{0\,a}$ / kJ·mol^{-1}	T_e (SD) / K	K_e / 10^4 M^{-1}	K_n / 10^2 M^{-1}	σ / 10^{-1}
1.0	–100.6 (2.0)	–5.6 (0.2)	–0.252 (0.007)	–25.4	305.9 (0.2)	10.0	11.1	1.1
2.0					311.4 (0.2)	5.0	5.8	1.2
3.0					314.7 (0.3)	3.3	3.9	1.2
4.0					317.1 (0.3)	2.5	3.0	1.2

[a] The Gibbs free energy was determined for a standard temperature of T = 298 K.

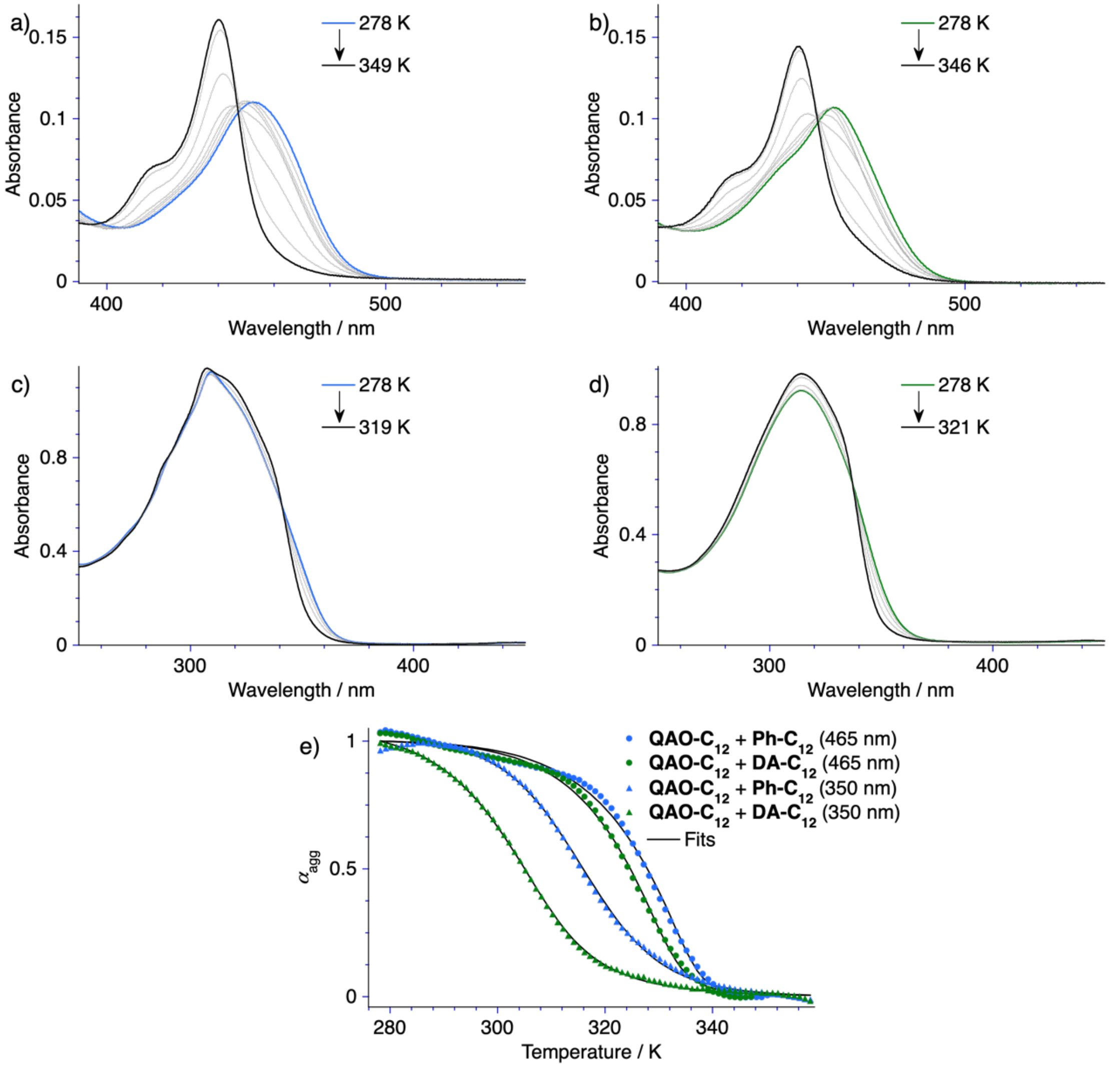

Figure S20. a-d) Temperature-dependent UV-vis absorption spectra of **QAO-C12** (c = 1.0 × 10^{-5} M) in the presence of **Ph-C12** (c = 1.0 × 10^{-4} M, a,c) and **DA-C12** (c = 1.0 × 10^{-4} M, b,d) in MCH between T = 278 K and T = 349 K using a heating rate of 1 K/min and a data interval of 1 K using a pathlength of l = 1 cm (a,b) and 1 mm (c,d) respectively. e) Aggregation parameter (α_{agg}) calculated based on the absorbance at λ_{abs} = 465 nm and 350 nm plotted against the temperature with cooperative fits derived from the nucleation-elongation model using an individual fitting approach.[7]

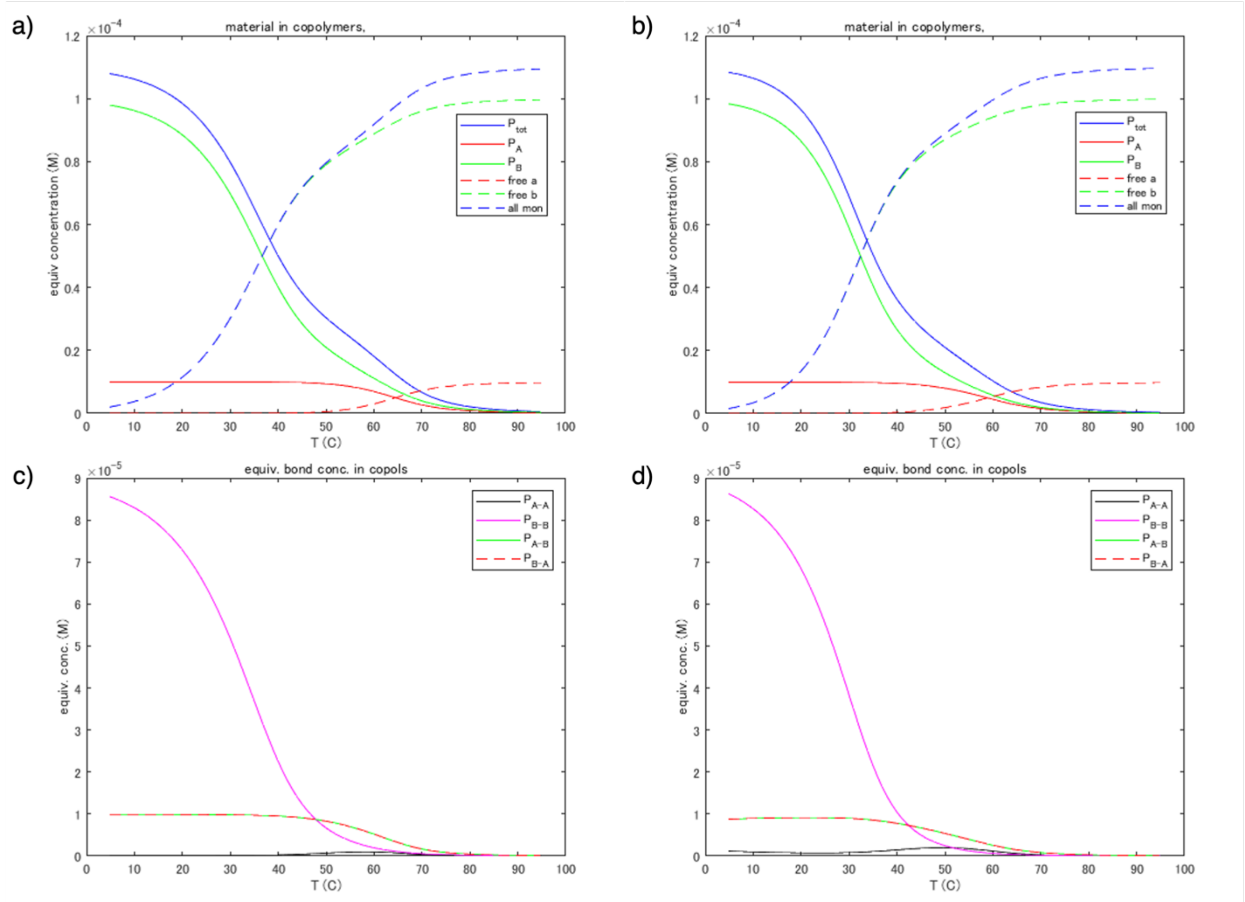

Figure S21. Equivalent concentrations (a,b) and equivalent bond concentrations (c,d) of the comonomers **QAO-C$_{12}$** (comonomer A) and **Ph-C$_{12}$** (comonomer B, a,c) and **QAO-C$_{12}$** (comonomer A) and **DA-C$_{12}$** (comonomer B, b,d) within the copolymer derived from the co-polymerization model based on the data shown in Figure S18.[10]

Table S7. Thermodynamic Parameters Derived from the Copolymerization Model Shown in Figure S19.[10]

Binding event	ΔH_e/ kJ mol^{-1}	ΔH_n/ kJ mol^{-1}	ΔS/ kJ mol^{-1} K^{-1}
**QAO-C$_{12}$** - **QAO-C$_{12}$**	–93.0	–9.0	–0.19
**Ph-C$_{12}$** - **Ph-C$_{12}$**	–80.0	–8.0	–0.18
**DA-C$_{12}$** - **DA-C$_{12}$**	–100.0	–6.0	–0.25
**QAO-C$_{12}$** - **Ph-C$_{12}$**	–88.0	–	–0.18
**QAO-C$_{12}$** - **DA-C$_{12}$**	–78.0	–	–0.16

Supplementary discussion 1

By analyzing the thermodynamic parameters in detail, the nanoscopic phase segregation behavior of the copolymers can be inferred. For the copolymer of **QAO-C$_{12}$** and **Ph-C$_{12}$** the difference in enthalpy between the binding events of **QAO-C$_{12}$** with itself compared to the binding event with **Ph-C$_{12}$** is 5 kJ·mol^{-1}, which means the binding with **Ph-C$_{12}$** comes at a minor enthalpic penalty (Table S7). For **Ph-C$_{12}$** the opposite holds true and the binding with **QAO-C$_{12}$** is accompanied by an enthalpic gain of 8 kJ·mol^{-1} (Table S7), likely due to an increase in aromatic interactions between **QAO-C$_{12}$** and **Ph-C$_{12}$**, that were found to be unfeasible for the homopolymer of **Ph-C$_{12}$** due to the rotational displacement.[4] In turn, this energetically favorable interaction leads to the efficient suppression of **QAO-C$_{12}$-QAO-C$_{12}$** homo-contacts within the copolymer structure, that is revealed in the simulated A-A (A referring to **QAO-C$_{12}$**) equivalent bond concentration below 45 °C (Figure S19c). Accordingly, the polymer adopts a random structure at low temperatures.

For the copolymer of **QAO-C$_{12}$** and **DA-C$_{12}$** a significantly larger difference in the binding enthalpy was unraveled, revealing an enthalpically penalty of 15 and 22 kJ·mol^{-1} between the binding event of **QAO-C$_{12}$** and **DA-C$_{12}$** with themselves and with each other (Table S7). This energetic penalty is directly reflected in the A-A equivalent bond concentration (A referring likewise to **QAO-C$_{12}$**), which stays above 0 for the entire investigated temperature range (Figure S19d). Given that the polymerization process is initiated by a **QAO-C$_{12}$** nucleus, the nanoscopic segregation behavior can be described as “blocky” with **QAO-C$_{12}$** dominating the central and **DA-C$_{12}$** dominating the peripheral regime.[10]

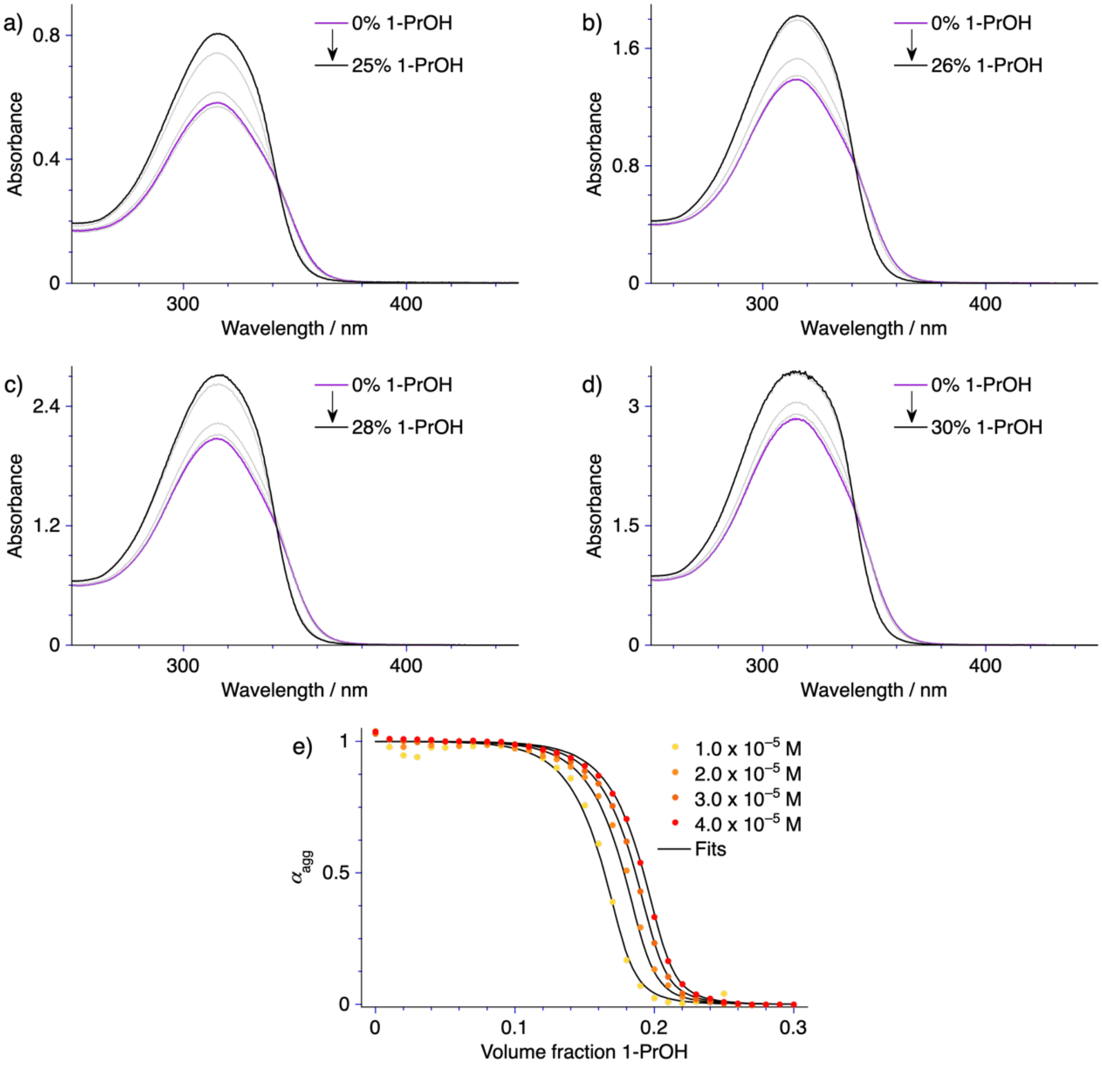

Figure S22. a-d) Solvent-dependent UV-vis absorption spectra of **DA-TEG** using water as self-assembly inducing and 1-PrOH as denaturing agent at T = 298 K and c = 1.0 × 10^{-5} M (a); c = 2.0 × 10^{-5} M (b); c = 3.0 × 10^{-5} M (c) and c = 4.0 × 10^{-5} M (d). e) Aggregation parameter (α_{agg}) calculated based on the absorbance at λ_{abs} = 355 nm plotted against the volume fraction of chloroform with cooperative fits derived from the solvent-dependent model using a global fitting approach.[6]

Table S8. Thermodynamic parameters derived from fitting the denaturation curves in Fig.S20 using water as self-assembly inducing and 1-PrOH as denaturing agent at T = 298 K to the solvent-dependent model using a global fitting approach.[6]

ΔG^0 / kJ·mol^{-1}	ΔG^0 (SD) / kJ·mol^{-1}	m / kJ·mol^{-1}	m (SD) / kJ·mol^{-1}	σ / 10^{-2}	σ (SD) / 10^{-3}
–49.6	4.6	122.0	2.7	5.7	8.0

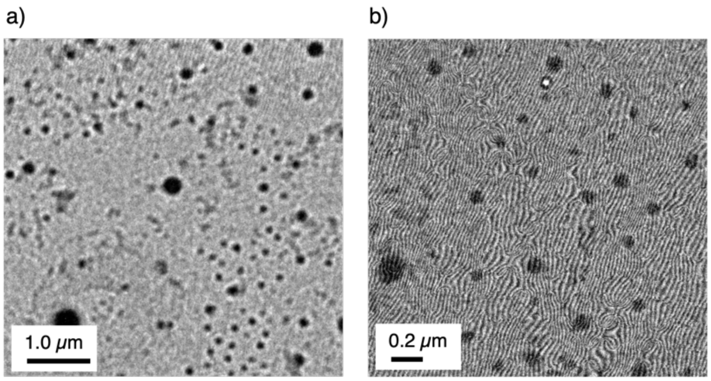

Figure S23. a,b) TEM micrographs of **DA-TEG** obtained from dropcasting (V = 20 μL) a water solution at c = 5.0 × 10^{-5} M. Scale bars correspond to 1.0 μm (a) and 0.2 μm (b).

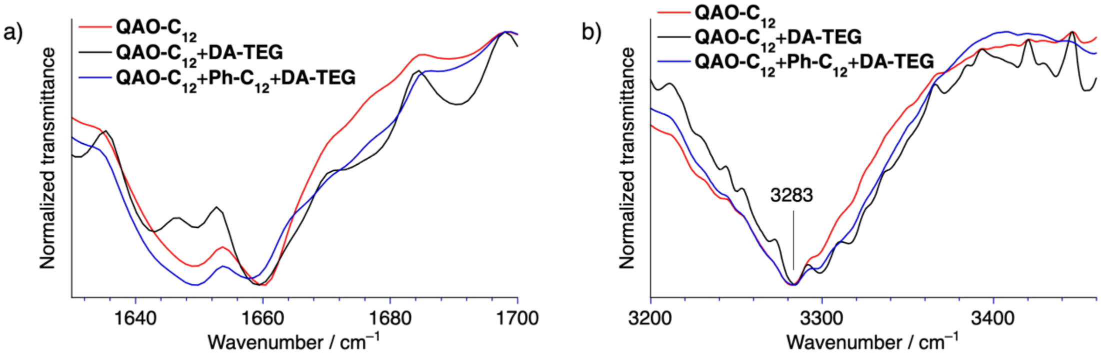

Figure S24. FT-IR spectra of **QAO-C$_{12}$**, **Ph-C$_{12}$**, and **DA-TEG** in the film state. Thin films were prepared by step wise dropcasting chloroform solutions (c = 1.0 x 10^{-3} M; V = 50 μL **QAO-C$_{12}$**; c = 5.0 x 10^{-4} M (each); V = 100 μL **QAO-C$_{12}$+DA-TEG**; c = 3.3 x 10^{-4} M (each) **QAO-C$_{12}$+Ph-C$_{12}$+DA-TEG**; V = 100 μL).

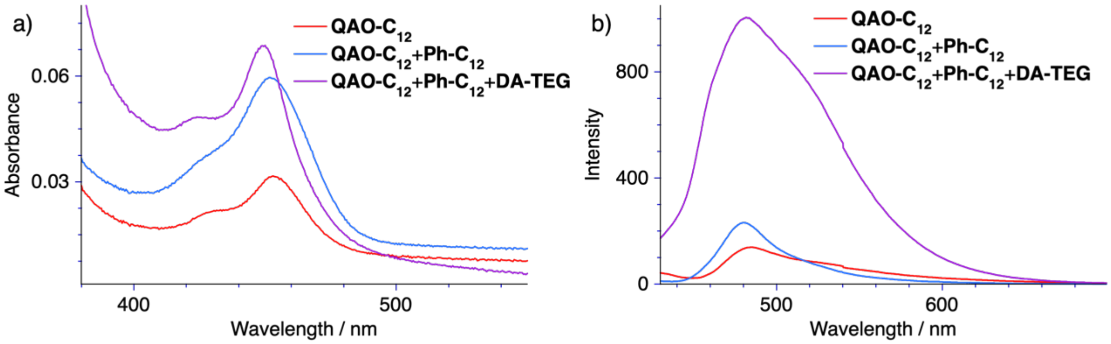

Figure S25. a,b) Thin-film UV-vis absorption (a) and photoluminescence (λ_{ex} = 405 nm, b) spectra of **QAO-C$_{12}$** obtained from dropcasting a total volume of V = 100μL of chloroform solutions of **QAO-C$_{12}$** (c = 2.5 × 10^{-5} M) in isolation, in a binary mixture with **Ph-C$_{12}$** (c = 1.0 × 10^{-4} M) and in a tertiary mixture with **Ph-C$_{12}$** (c = 1.0 × 10^{-4} M) and **DA-TEG** (c = 2.5 × 10^{-3} M).

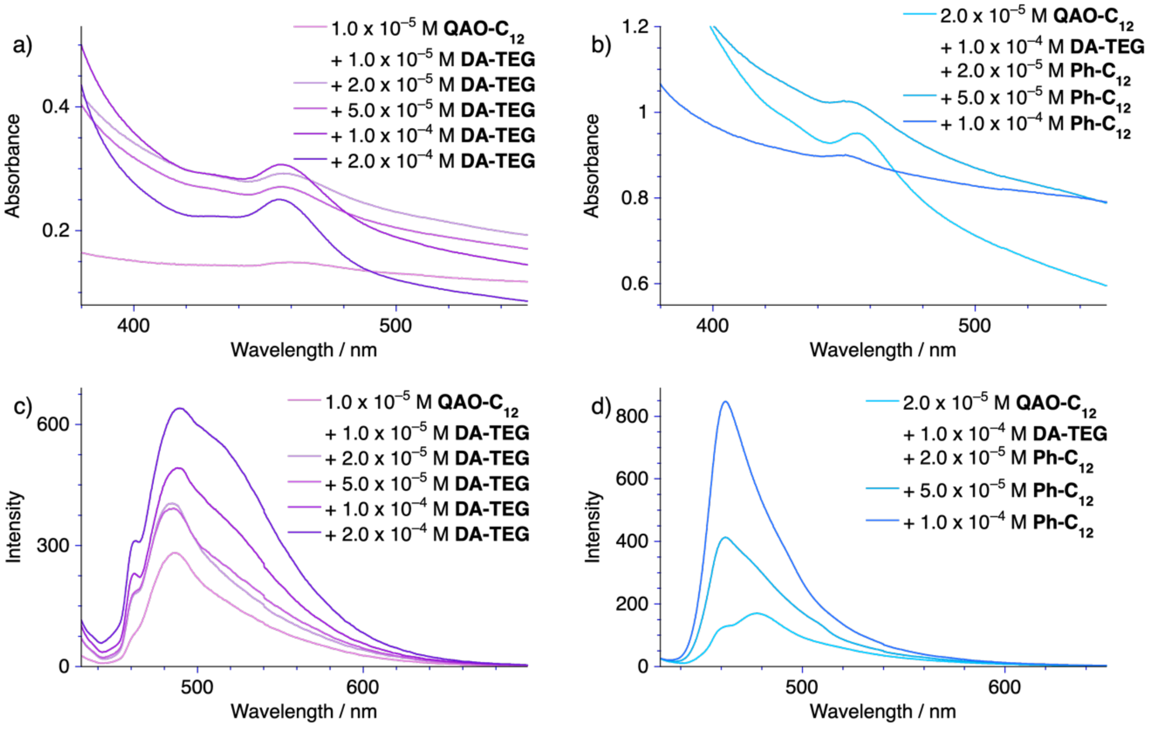

Figure S26. a-d) UV-vis (a,b) and photoluminescence (c,d; λ_{ex} = 430 nm) spectra of **QAO-C12** at c = 1.0 x 10^{-5} M and T = 298 K in the presence of varying amounts of **DA-TEG** (a,c) and of **QAO-C12** at c = 2.0 x 10^{-5} M and **DA-TEG** at c = 1.0 x 10^{-4} M and T = 298 K in the presence of varying amounts of **Ph-C12** (b,d) in water.

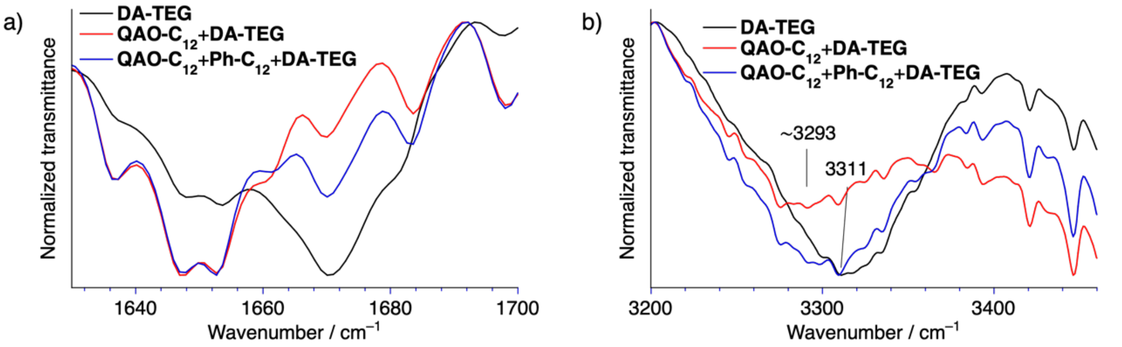

Figure S27. FT-IR spectra of **QAO-C12**, **Ph-C12**, and **DA-TEG** in the film state. Thin films were prepared by step wise dropcasting water solutions (c = 5.0 x 10^{-4} M; V = 100 μL **DA-TEG**; c = 5.0 x 10^{-4} M (each); V = 100 μL **QAO-C12+DA-TEG**; c = 5.0 x 10^{-4} M **DA-TEG** and c = 2.5 x 10^{-4} M (each) **QAO-C12+Ph-C12**; V = 100 μL).

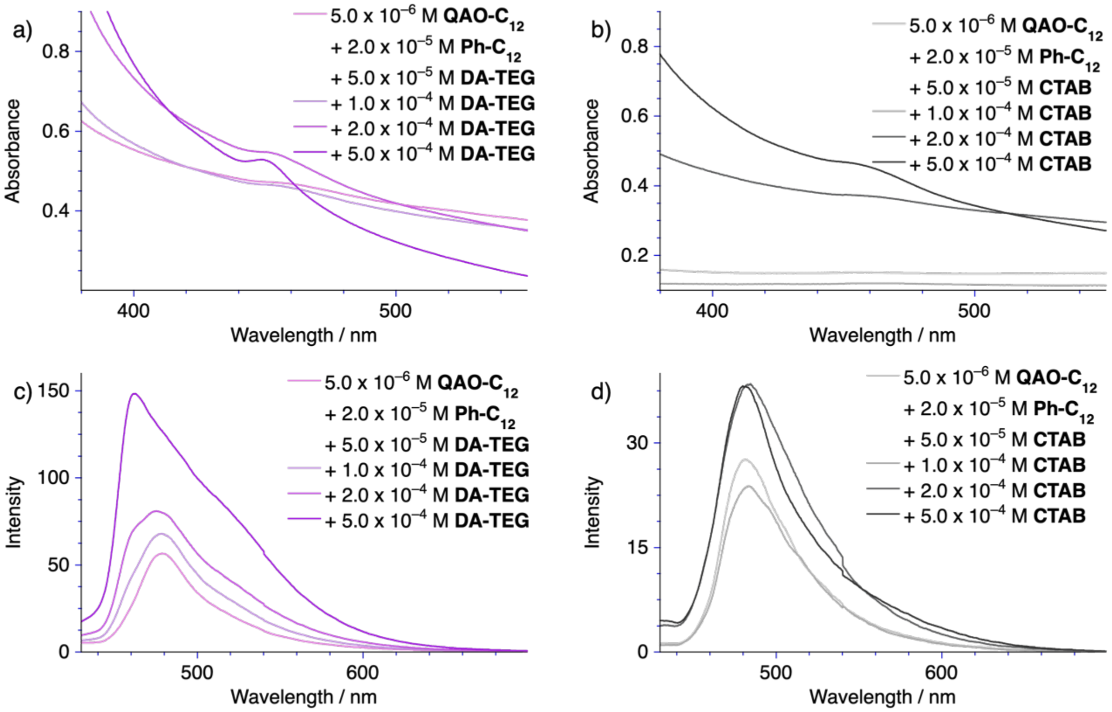

Figure S28. a-d) UV-vis (a,b) and photoluminescence (c,d; λ_{ex} = 405 nm) spectra of **QAO-C$_{12}$** at *c* = 5.0 x 10^{-6} M and **Ph-C$_{12}$** at *c* = 2.0 x 10^{-5} M at *T* = 298 K in the presence of varying amounts of **DA-TEG** (a,c) and cetrimonium bromide (**CTAB**; b,d) in water.

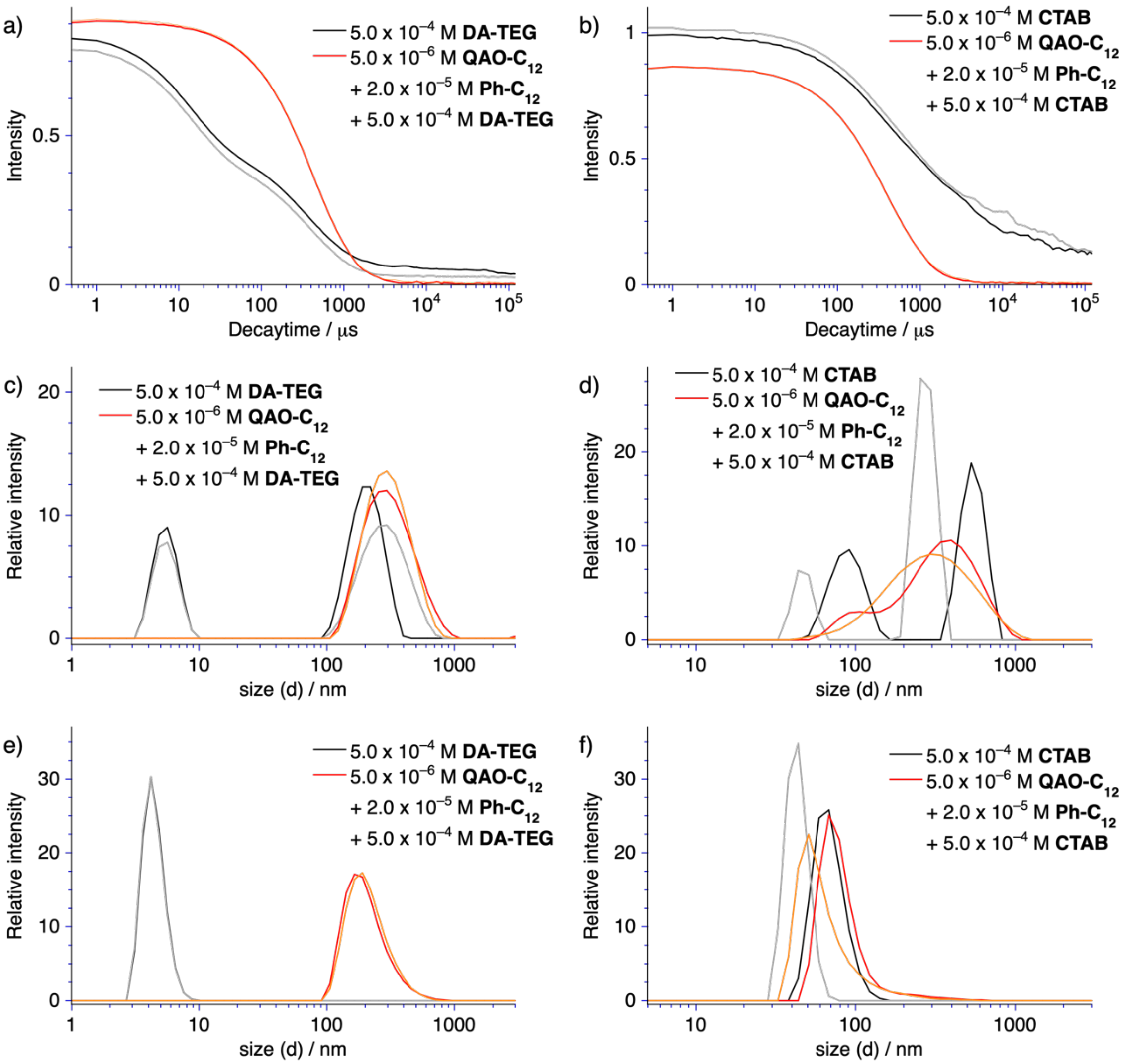

Figure S29. a-f) DLS correlation (a,b), intensity size distribution (c,d) and number size distribution (e,f) function of **QAO-C12** at c = 5.0 x 10^{-6} M and **Ph-C12** at c = 2.0 x 10^{-5} M at T = 298 K in the presence of **DA-TEG** (a,c,e) and **CTAB** (b,d,f) at c = 5.0 x 10^{-4} M in water with the spectra of **DA-TEG** and **CTAB** at c = 5.0 x 10^{-4} M in water shown for reference.

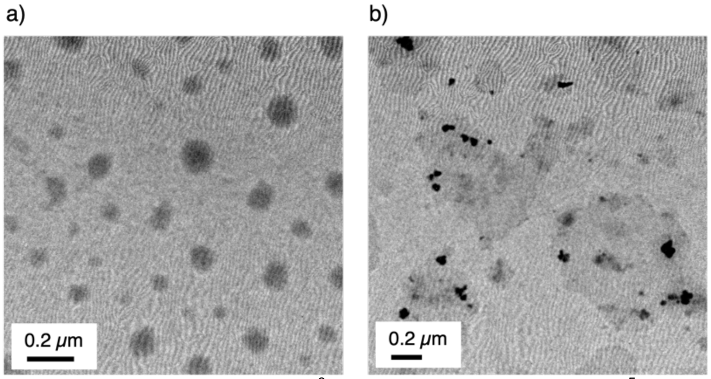

Figure S30. TEM micrographs of **QAO-C12** at c = 5.0 x 10^{-6} M and **Ph-C12** at c = 2.0 x 10^{-5} M at T = 298 K in the presence of **DA-TEG** (a) and **CTAB** (b) at c = 5.0 x 10^{-4} M obtained from dropcasting (V = 20 μL) a water solution. Scale bars correspond to 0.2 μm.

Supplementary discussion 2

The molecular design of the individual constituents of the three-component system is key to balancing the overall solubilization, the photoluminescence brightness and the associated band shape. Specifically, **QAO-C$_{12}$** bestows the system with an MR-TADF chromophore. However, the strong solvophobic effects in water lead to low emission intensities and large FWHM even in the presence of **DA-TEG** as a solubilizing agent (Figure S25a). To circumvent these undesirable photoluminescence properties, **Ph-C$_{12}$** is used as a modulator, reinstating a narrower band shape and increased photoluminescence brightness (Figure S25b), akin to co-dopant strategies employed in OLEDs.[11,12] Crucially, the molecular structure of the solubilizing agent was found to have a profound influence on the photoluminescence properties as well as the solubilization. While screening commercially available surfactants, we found that sodium dodecyl sulfate (**SDS**) and Triton™ X-100 were completely unsuitable for the solubilization of the copolymer of **QAO-C$_{12}$** and **Ph-C$_{12}$**, resulting in large, poorly dissolved clusters of organic material. In contrast, **CTAB,** which has a longer aliphatic group compared to **SDS** and Triton™ X-100, could achieve a moderate level of solubilization. Yet, the achieved photoluminescence intensity remained low, even at high concentrations of the surfactant (Figure S27). We reasoned that a high degree of molecular similarity between the solubilizing agent and the supramolecular synthons may be a prerequisite to simultaneously improve the overall solubility and photoluminescence profiles. Based on increased photoluminescence upon increasing the concentration of **DA-TEG** (Figure S27a), we deduce that a fraction of **DA-TEG** must be incorporated into the stacks of **QAO-C$_{12}$** and **Ph-C$_{12}$** upon sample preparation, resulting in a more polar copolymer. Consequently, the incorporated **DA-TEG** may serve as a binding side for recruiting additional **DA-TEG** towards the formation of a solubilizing shell existing exclusively of **DA-TEG** (core-shell structure inset in Figure 4b). This hypothesis appears to be validated by TEM analysis, revealing relatively homogeneous structures for the tertiary system of **QAO-C$_{12}$**, **Ph-C$_{12}$** and **DA-TEG**, compared to the highly inhomogeneous structures in the presence of **CTAB**, as solubilizing agent (Figure S28).

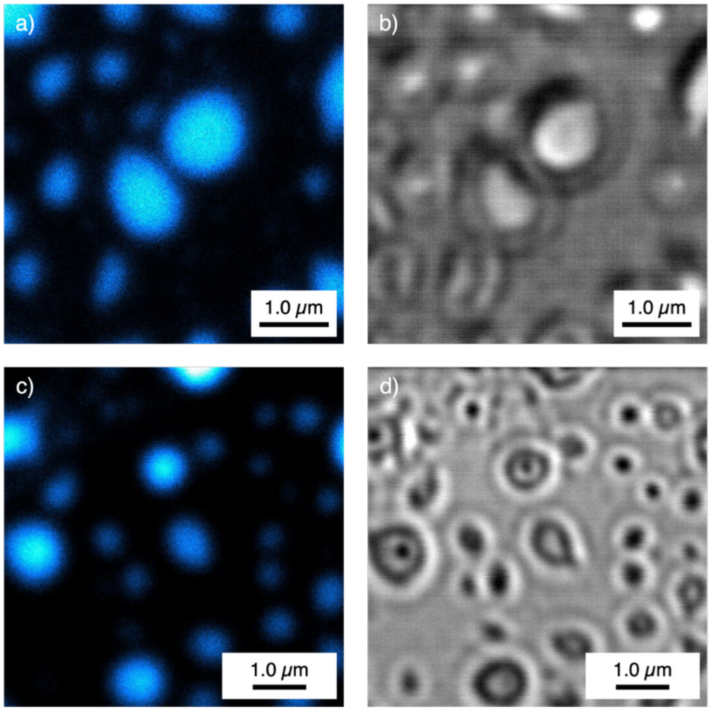

Figure S31. a-d) Fluorescence (a,c) and confocal microscopy (b,d) images of **QAO-C$_{12}$** at *c* = 5.0 x 10^{-7} M, **Ph-C$_{12}$** at *c* = 2.0 x 10^{-6} M and **DA-TEG** at *c* = 5.0 x 10^{-5} M at T = 298 K (λ_{ex} = 420 nm, λ_{em} = 450–550 nm). The samples were obtained from drying water solutions with **QAO-C$_{12}$** at *c* = 5.0 x 10^{-6} M, **Ph-C$_{12}$** at *c* = 2.0 x 10^{-5} M and **DA-TEG** at *c* = 5.0 x 10^{-4} M that were diluted with water in a 1:10 ratio immediately prior to dropcasting.

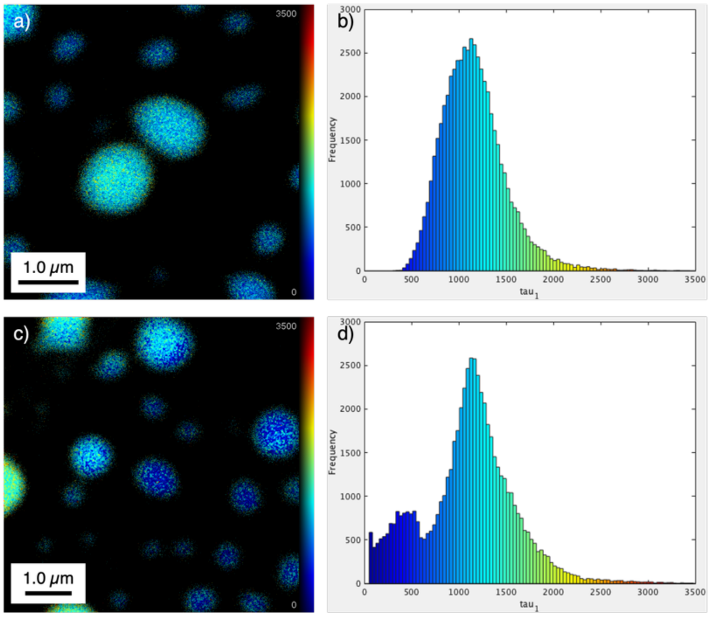

Figure S32. a-d) Fluorescence lifetime microscopy image (a,c) and lifetime histogram (b,d) of **QAO-C12** at c = 5.0 x 10^{-7} M, **Ph-C12** at c = 2.0 x 10^{-6} M and **DA-TEG** at c = 5.0 x 10^{-5} M at T = 298 K (λ_{ex} = 420 nm, λ_{em} = 450–550 nm). The samples were obtained from drying water solutions with **QAO-C12** at c = 5.0 x 10^{-6} M, **Ph-C12** at c = 2.0 x 10^{-5} M and **DA-TEG** at c = 5.0 x 10^{-4} M that were diluted with water in a 1:10 ratio immediately prior to dropcasting.

References

[1] Gaussian 16, Revision C.01, M. J. Frisch, G. W. Trucks, H. B. Schlegel, G. E. Scuseria, M. A. Robb, J. R. Cheeseman, G. Scalmani, V. Barone, G. A. Petersson, H. Nakatsuji, X. Li, M. Caricato, A. V. Marenich, J. Bloino, B. G. Janesko, R. Gomperts, B. Mennucci, H. P. Hratchian, J. V. Ortiz, A. F. Izmaylov, J. L. Sonnenberg, D. Williams-Young, F. Ding, F. Lipparini, F. Egidi, J. Goings, B. Peng, A. Petrone, T. Henderson, D. Ranasinghe, V. G. Zakrzewski, J. Gao, N. Rega, G. Zheng, W. Liang, M. Hada, M. Ehara, K. Toyota, R. Fukuda, J. Hasegawa, M. Ishida, T. Nakajima, Y. Honda, O. Kitao, H. Nakai, T. Vreven, K. Throssell, J. A. Montgomery, Jr., J. E. Peralta, F. Ogliaro, M. J. Bearpark, J. J. Heyd, E. N. Brothers, K. N. Kudin, V. N. Staroverov, T. A. Keith, R. Kobayashi, J. Normand, K. Raghavachari, A. P. Rendell, J. C. Burant, S. S. Iyengar, J. Tomasi, M. Cossi, J. M. Millam, M. Klene, C. Adamo, R. Cammi, J. W. Ochterski, R. L. Martin, K. Morokuma, O. Farkas, J. B. Foresman, D. J. Fox, Gaussian, Inc., Wallingford CT, 2019.
[2] (a) M. Cossi, N. Rega, G. Scalmani, V. Barone, *J. Comput. Chem.* **2003**, 24, 669–681; (b) J. Tomasi, B. Mennucci, R. Cammi, *Chem. Rev.* **2005**, 105, 2999.
[3] X. Qiu, G. Tian, C. Lin, Y. Pan, X. Ye, B. Wang, D. Ma, D. Hu, Y. Luo, Y. Ma, *Adv. Opt. Mater.* **2021**, *9*, 2001845.
[4] N. Bäumer, S. Yamada, S. Ogi, S. Yamaguchi, *J. Am. Chem. Soc.*, **2025**, *147*, 8300–8311.
[5] S. Das, D. Patra, S. Shankar, A. Ajayaghosh, *Angew. Chem. Int. Ed.* **2022**, *61*, e202207641.
[6] P. A. Korevaar, C. Schaefer, T. F. A. de Greef, E. W. Meijer, *J. Am. Chem. Soc.* **2012**, *134*, 13482.
[7] H. M. M. ten Eikelder, A. J. Markvoort, T. F. A. de Greef, P. A. J. Hilbers, *J. Phys. Chem. B* **2012**, *116*, 5291.
[8] Y. Tsuchiya, S. Diesing, F. Bencheikh, Y. Wada, P. L. dos Santos, H. Kaji, E. Zysman-Colman, I. D. W. Samuel, C. Adachi, *J. Phys. Chem. A*, **2021**, *125*, 8074–8089.
[9] N. Bäumer, S. Ogi, S. Yamaguchi, *Angew. Chem. Int. Ed.*, **2025**, *64*, e202501693.
[10] H. M. M. ten Eikelder, B. Adelizzi, A. R. A. Palmans, A. J. Markvoort, *J. Phys. Chem. B* **2019**, *123*, 6627-6642.
[11] H. Nakanotani, T. Higuchi, T. Furukawa, K. Masui, K. Morimoto, M. Numata, H. Tanaka, Y. Sagara, T. Yasuda, C. Adachi, *Nat. Commun.* **2014**, *5*, 4016.
[12] S. O. Jeon, K. H. Lee, J. S. Kim, S.-G. Ihn, Y. S. Chung, J. W. Kim, H. Lee, S. Kim, H. Choi, J. Y. Lee, *Nat. Photon.* **2021**, *15*, 208–215.

Computational section

Table S9. Cartesian coordinates of the optimized dimer structure of **QAO-Me**. Calculations were performed at the M052X/6-31G(d,p) level of theory with methylcyclohexane as solvent using the polarizable continuum model.

atom	x	y	z	atom	x	y	z
C	2.8275493	-3.1217129	-1.4224813	H	-11.912244	-4.0297264	1.8921801
C	1.9467839	-2.2978899	-1.5330408	C	-12.953754	-9.1256049	2.6824431
C	0.8998084	-1.3290422	-1.6474943	H	-13.033065	-8.3802229	3.4754402
C	-1.1514768	0.5558372	-1.8243987	H	-12.239513	-9.9014334	2.9639531
C	-0.4366008	-1.7452978	-1.6419665	H	-13.927899	-9.5714203	2.4957121
C	1.2103335	0.0318636	-1.7481942	C	-9.21953	-10.645822	0.461481
C	0.182798	0.9753097	-1.8318165	H	-8.5148938	-10.713333	1.2933329
C	-1.4657338	-0.8037321	-1.7356671	H	-8.7216643	-10.202556	-0.4038718
H	-0.6724215	-2.797885	-1.5665873	H	-9.5804265	-11.638061	0.2059757
H	2.2434534	0.3497994	-1.7349422	C	11.4908184	-5.9987485	2.6824778
H	-1.9446152	1.2883751	-1.8826741	H	11.332982	-5.1488986	3.3504714
C	-2.8400271	-1.2029502	-1.7458992	H	11.8389656	-6.8539085	3.2548142
C	-4.0186113	-1.4812312	-1.7589947	H	10.5603601	-6.2478207	2.1685939
C	0.4555963	2.378832	-1.9036593	C	15.4179645	-4.8715612	0.6705155
C	0.5915324	3.5808651	-1.9545986	H	15.0798484	-4.8113265	1.7062802
C	-5.4282631	-1.7225568	-1.767923	H	15.7397695	-3.8889215	0.3210926
C	-8.187793	-2.12682	-1.7520626	H	16.2402917	-5.578637	0.5910037
C	-5.9543178	-3.0092454	-1.5977796	C	14.0451722	-2.4381336	-2.9843096
C	-6.3038764	-0.639911	-1.9348767	H	13.9590218	-1.4483378	-2.5306188
C	-7.6743857	-0.8417729	-1.9217329	H	13.1853072	-2.6188597	-3.6331426
C	-7.3277496	-3.2123026	-1.5949575	H	14.9602384	-2.4956035	-3.5668666
H	-5.2817622	-3.8442509	-1.4552734	O	-6.4893053	-6.6858763	1.8234439
H	-5.8981169	0.3547266	-2.0629157	O	9.8296526	-1.0088117	-0.7637169
H	-8.3538131	-0.0060705	-2.0361199	O	-14.544117	-1.1257064	-2.7403468
H	-7.7369398	-4.2049407	-1.4637066	O	-14.542446	-5.5087665	-1.2026792
C	3.8547597	-4.1010443	-1.250307	O	-15.802083	-3.4041731	-2.221783
C	5.8630124	-5.9841469	-0.7918641	C	-13.902561	0.1125903	-3.001965
C	5.1978547	-3.7778393	-1.4772044	H	-14.656893	0.7495205	-3.4545767
C	3.5266817	-5.3934789	-0.8163955	H	-13.068396	-0.0154728	-3.6966634
C	4.5246184	-6.324405	-0.5835991	H	-13.544481	0.5718352	-2.0776345
C	6.198378	-4.7146758	-1.2598875	C	-16.027227	-3.9476364	-3.5252739
H	5.4558285	-2.7781726	-1.8018829	H	-17.105243	-3.9855117	-3.6621963
H	2.4898469	-5.6495	-0.6439358	H	-15.610456	-4.953869	-3.5928791
H	4.2734393	-7.3141369	-0.2208805	H	-15.581308	-3.3027578	-4.2854013
H	7.2339888	-4.4609074	-1.4333939	C	-13.93298	-6.6242076	-0.5516885
N	6.8443279	-6.9743275	-0.5533611	H	-14.687336	-7.4049123	-0.5337272
H	6.6119792	-7.9160934	-0.8226022	H	-13.642325	-6.3726235	0.4664292
N	-9.5960136	-2.3074605	-1.8088402	H	-13.062141	-6.9754269	-1.1099925
H	-10.072996	-1.9551172	-2.6225505	O	8.4630588	-11.624457	0.9957172
C	-10.344592	-2.9888222	-0.9034346	O	11.0702626	-11.531748	0.5046483
C	8.0661316	-6.7927075	0.0182825	O	12.2715656	-9.3096692	-0.3070614
C	-11.800949	-3.0645195	-1.2407044	C	7.0802443	-11.692104	1.3066815
C	-14.476206	-3.3019373	-1.9178598	H	6.8374966	-11.049372	2.1561335
C	-12.461204	-4.2616603	-0.9733834	H	6.8837369	-12.727876	1.5677
C	-12.452165	-1.9788726	-1.8293138	H	6.4687252	-11.410916	0.4454602
C	-13.800695	-2.102756	-2.1676687	C	11.1937673	-12.254081	-0.723497
C	-13.804327	-4.3888037	-1.3410451	H	10.2090492	-12.562382	-1.081063
H	-11.914552	-5.0732177	-0.5132704	H	11.7968915	-13.132239	-0.5058506
H	-11.930564	-1.0440693	-1.9769432	H	11.6926252	-11.640223	-1.475166
C	8.8518785	-8.0585726	0.1742884	C	12.9949024	-8.1086295	-0.5781459
C	10.3353642	-10.39041	0.371801	H	12.9640272	-7.4359566	0.2763857
C	10.2202184	-8.0114795	-0.0831714	H	12.6021909	-7.6059362	-1.4651469
C	8.2121281	-9.2443972	0.5420256	H	14.0196462	-8.4160484	-0.7634161
C	8.9636257	-10.416059	0.6432087	H	-8.3623746	-4.2352115	1.1657016
C	10.9609821	-9.1959188	-0.0124247	C	0.6162643	5.0101148	-1.9773032
H	10.673781	-7.0689476	-0.3575215	C	0.542939	7.8366864	-2.0334493
H	7.1602838	-9.2426339	0.7894099	C	-0.5140537	5.7089238	-1.543852
O	-9.8761461	-3.523307	0.0993573	C	1.7181978	5.7455048	-2.4208756
O	8.5053282	-5.697437	0.3596962	C	1.6870171	7.135791	-2.4613306
C	3.594719	-0.3049745	1.2272933	C	-0.5547301	7.0957835	-1.5549269
C	2.5078459	0.2064915	1.3860072	H	-1.382548	5.1818213	-1.1727234
C	1.227974	0.8286234	1.5431964	H	2.6254921	5.2542064	-2.7469539
C	-1.2795348	2.0602413	1.7824492	C	-1.7471532	7.7683468	-1.0159994
C	0.0686532	0.0523863	1.6309605	C	2.8733885	7.861125	-2.9517393
C	1.1296804	2.2217524	1.5775374	O	3.933031	7.3067902	-3.1919658
C	-0.1210115	2.8372457	1.702277	O	-2.7635573	7.1626577	-0.7095214

C	-1.1847585	0.6637458	1.7438497	C	2.6282476	9.2840603	-3.2563066
H	0.1378576	-1.0258316	1.5880499	C	2.0774853	11.8278795	-4.211015
H	2.0214738	2.8284203	1.5029644	C	3.5725496	9.9692247	-4.0272803
H	-2.2468306	2.5352426	1.8709041	C	1.4210023	9.9058796	-2.9017565
C	-2.3525132	-0.1608422	1.7900021	C	1.1324021	11.1666464	-3.4444438
C	-3.2991077	-0.9172446	1.7954458	C	3.3204597	11.2494664	-4.4820518
C	-4.3698535	-1.8624842	1.7694352	H	4.4878937	9.4437567	-4.2665897
C	-6.4198238	-3.7706026	1.6274228	H	0.16436	11.6172656	-3.285485
C	-4.0843015	-3.2319126	1.8293087	H	4.0530359	11.7774839	-5.0778216
C	-5.7076561	-1.4582315	1.6526461	H	1.8316871	12.800089	-4.6191299
C	-6.7170662	-2.4007645	1.586892	C	-1.5764254	9.2114436	-0.7842576
C	-5.0899307	-4.1850566	1.7617066	C	-1.206656	11.8596303	-0.0720505
H	-3.053344	-3.5486604	1.9206423	C	-0.4558583	9.9015922	-1.2786676
H	-5.9445231	-0.4037395	1.5999281	C	-2.5080871	9.8595347	0.0337662
H	-7.748308	-2.0887295	1.470764	C	-2.3400671	11.1841552	0.3868336
H	-4.8598203	-5.2366203	1.7993771	C	-0.2749455	11.2378873	-0.8877458
C	4.8836037	-0.8838705	1.0064787	H	-3.3394605	9.2710365	0.3991545
C	7.4072237	-2.0052718	0.5114027	H	-3.0534669	11.6779658	1.0330031
C	5.8369409	-0.20055	0.2417054	H	0.6126124	11.7781844	-1.1791915
C	5.2182794	-2.1385938	1.5348469	H	-1.0279189	12.8848909	0.2283135
C	6.4651993	-2.6865821	1.2943115	N	0.4955144	9.2252514	-2.0764562
C	7.0873573	-0.7465843	-0.0117678	C	-0.1683681	4.2686792	1.7465837
H	5.5871752	0.7705201	-0.1660436	C	-0.0997827	5.4769339	1.7962951
H	4.4902375	-2.681089	2.1233272	C	0.1175176	6.8896085	1.8475173
H	6.7128775	-3.6671075	1.6840347	C	0.7325239	9.6416605	2.0578787
H	7.8100326	-0.2163415	-0.6086624	C	-0.7163462	7.755393	2.5599475
N	8.6304071	-2.6558123	0.2856242	C	1.2205237	7.4337645	1.1812097
H	8.640515	-3.64893	0.4883111	C	1.5202922	8.7880898	1.2617686
N	-7.5036115	-4.6522088	1.5095736	C	-0.4133881	9.1081686	2.6783846
C	-7.4981619	-6.0099551	1.6675867	H	-1.6113015	7.3925504	3.0481383
C	9.7655946	-2.1222774	-0.2604087	H	1.8711991	6.8062539	0.5848252
C	-8.8574312	-6.6416906	1.6349791	C	2.6508897	9.3155244	0.4834691
C	-11.325674	-7.9000435	1.554438	C	-1.3156059	9.9826858	3.4471146
C	-8.927753	-7.9748624	1.236287	C	2.7475764	10.7844305	0.4611261
C	-9.991708	-5.9397565	2.0334845	C	2.744877	13.5318479	0.1036368
C	-11.230451	-6.5771448	1.9985654	C	1.9401077	11.5820633	1.2892859
C	-10.168914	-8.6066843	1.1965919	C	3.5713122	11.3727302	-0.5068045
H	-8.010312	-8.4812262	0.9765224	C	3.5850879	12.7414821	-0.6868358
H	-9.9118133	-4.9218441	2.3798609	C	1.9331173	12.9705003	1.0751643
C	10.9692847	-3.0131882	-0.2006563	H	4.1646798	10.7082646	-1.1212113
C	13.2607891	-4.5706533	-0.1546138	H	4.2119881	13.1909628	-1.4450141
C	11.9645073	-2.7868253	-1.1489086	H	1.2635442	13.6050591	1.6351418
C	11.1133168	-3.9800139	0.7909737	H	2.7068987	14.6029712	-0.0502082
C	12.2706429	-4.7561015	0.8159091	C	-0.7060299	11.261318	3.8574532
C	13.1162267	-3.5700506	-1.1254381	C	0.5877581	13.4566613	4.9491073
H	11.8143732	-1.9974793	-1.8698866	C	-1.2947817	11.9786713	4.9040984
H	10.352715	-4.1144974	1.5434707	C	0.5141088	11.6853227	3.3095566
O	-12.398174	-5.9938278	2.3754959	C	1.1804996	12.7658552	3.9050229
O	-12.548527	-8.5045336	1.4583927	C	-0.6679937	13.0873765	5.4393508
O	-10.369225	-9.8956384	0.8262152	H	-2.2377107	11.6112639	5.2875182
O	14.3687245	-5.3715994	-0.1645003	H	2.1681855	13.0453753	3.5690684
O	12.5257007	-5.7148051	1.7440736	H	-1.1219041	13.6384327	6.2519351
O	14.1530826	-3.4475004	-1.9902042	H	1.1223958	14.2836519	5.3987141
C	-12.351473	-4.6078746	2.7076016	N	1.0802996	10.9748384	2.2278077
H	-11.781596	-4.4506993	3.6261621	O	-2.4361175	9.6397484	3.7917393
H	-13.383872	-4.3075486	2.8627552	O	3.3921525	8.5949045	-0.1715306

NMR spectra

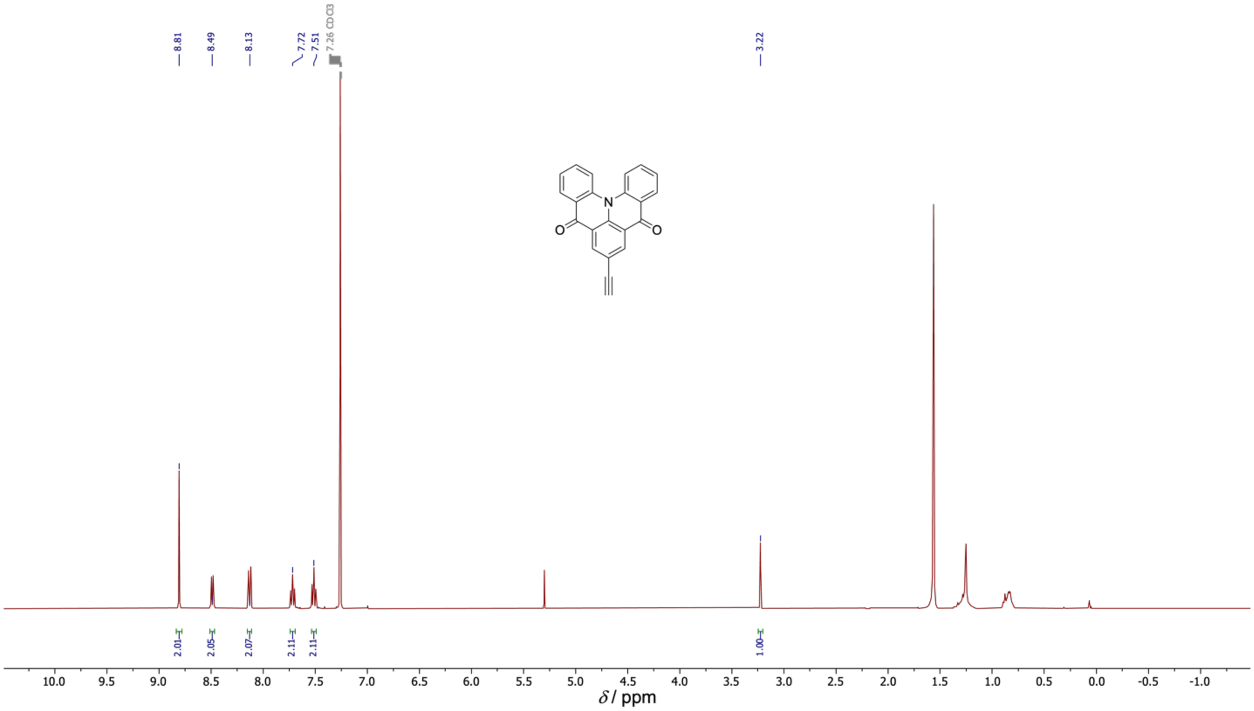

Figure S33. ^{1}H NMR spectrum of compound **4** (400 MHz, 298 K, $CDCl_3$).

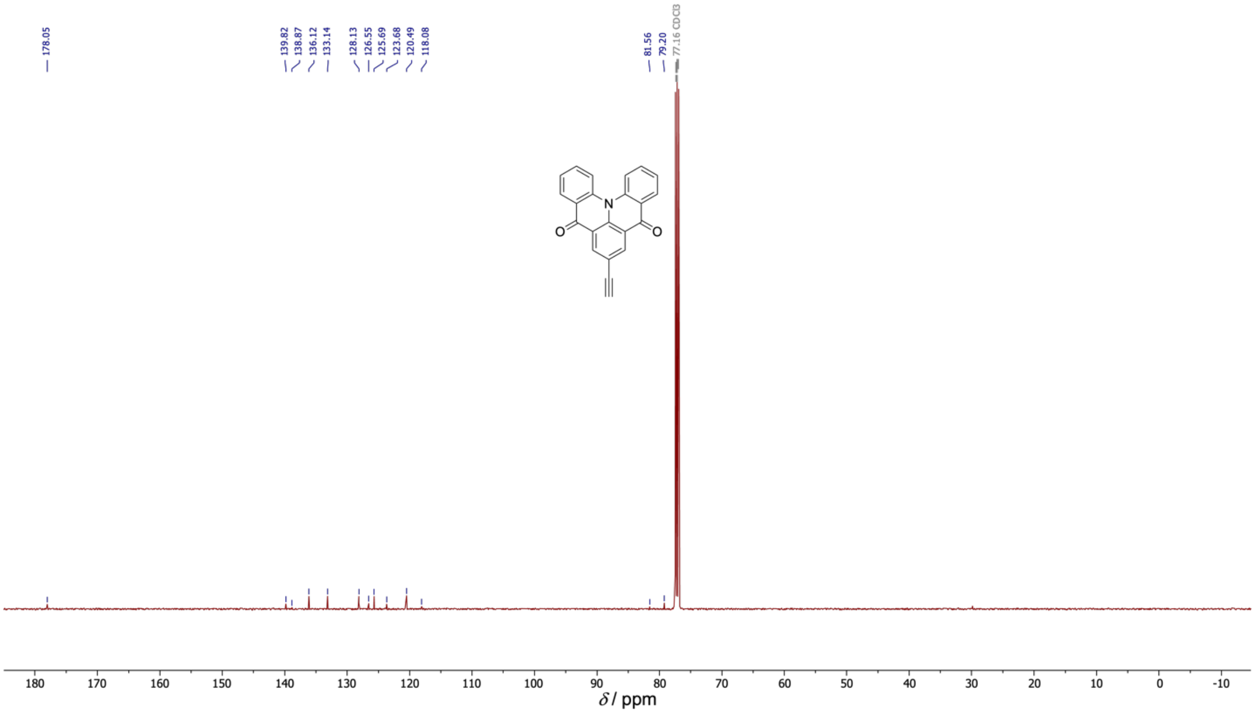

Figure S34. ^{13}C NMR spectrum of compound **4** (100 MHz, 298 K, $CDCl_3$).

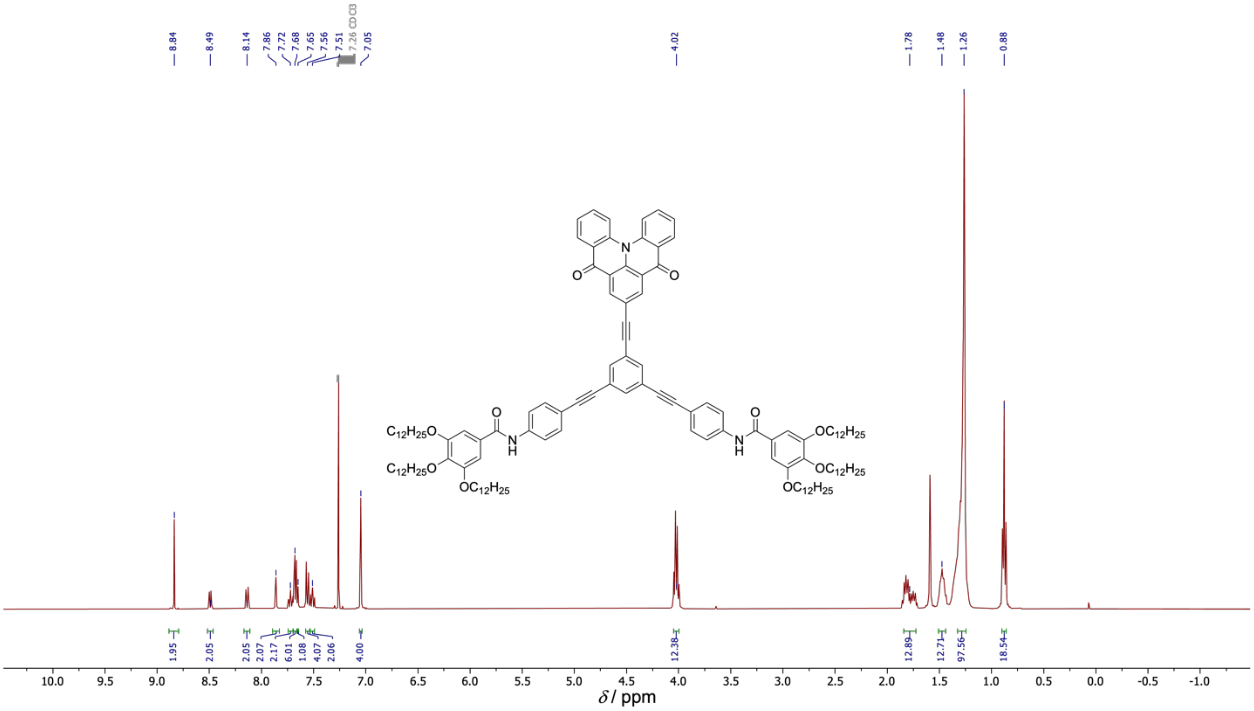

Figure S35. ^{1}H NMR spectrum of compound **QAO-C$_{12}$** (400 MHz, 298 K, $CDCl_3$).

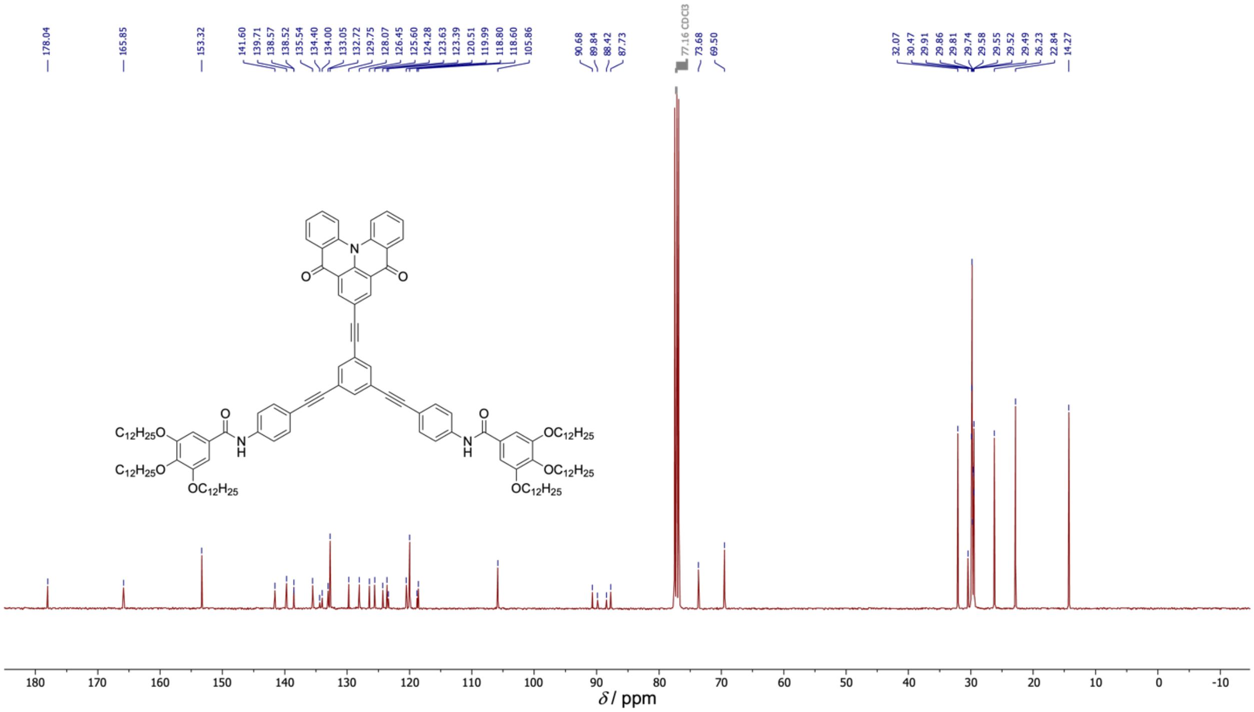

Figure S36. ^{13}C NMR spectrum of compound **QAO-C$_{12}$** (100 MHz, 298 K, $CDCl_3$).

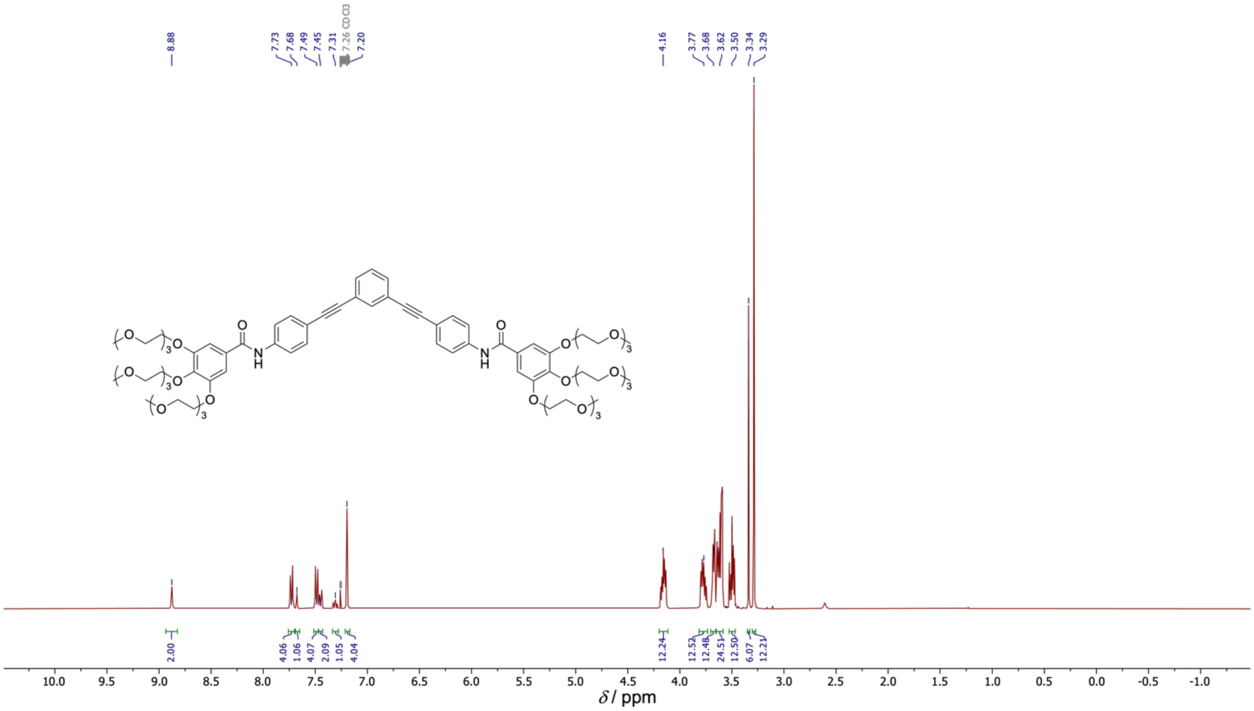

Figure S37. ^{1}H NMR spectrum of compound **DA-TEG** (400 MHz, 298 K, $CDCl_3$).

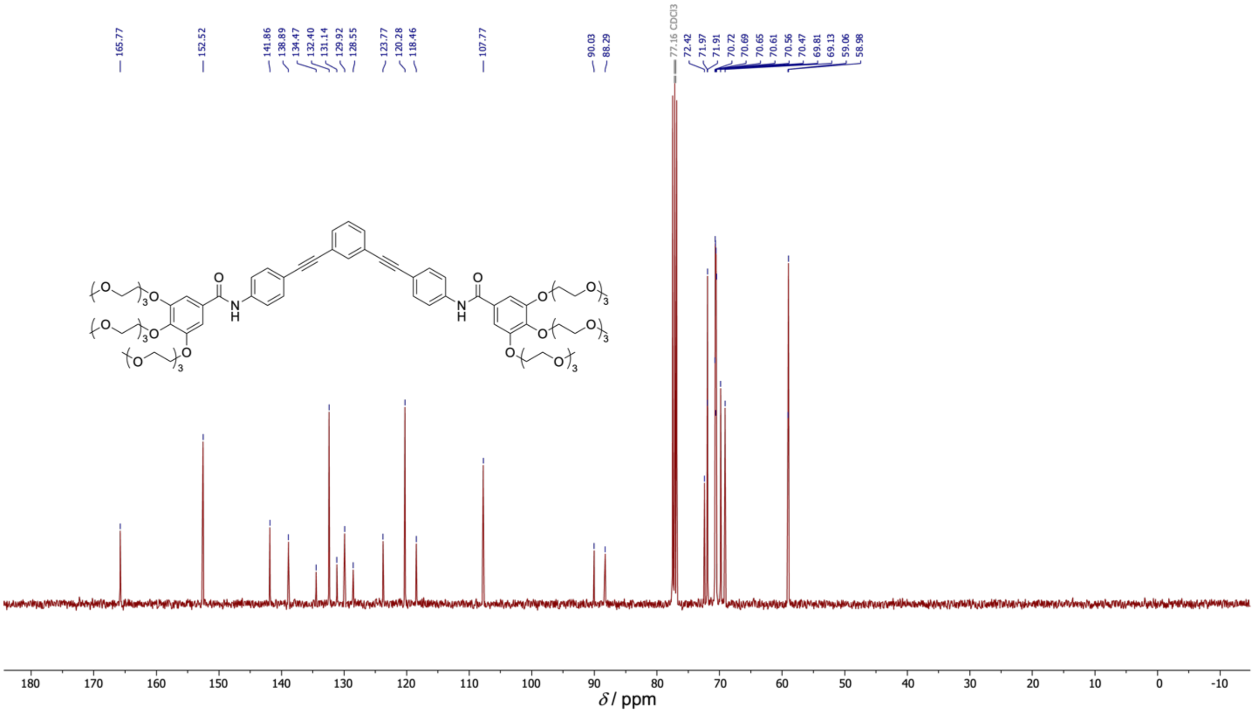

Figure S38. ^{13}C NMR spectrum of compound **DA-TEG** (100 MHz, 298 K, $CDCl_3$).
